# Predicting neutralization susceptibility to combination HIV-1 monoclonal broadly neutralizing antibody regimens

**DOI:** 10.1371/journal.pone.0310042

**Published:** 2024-09-06

**Authors:** Brian D. Williamson, Liana Wu, Yunda Huang, Aaron Hudson, Peter B. Gilbert

**Affiliations:** 1 Biostatistics Division, Kaiser Permanente Washington Health Research Institute, Seattle, WA, United States of Amerrica; 2 Vaccine and Infectious Disease Division, Fred Hutchinson Cancer Center, Seattle, WA, United States of Amerrica; 3 Department of Biostatistics, University of Washington, Seattle, WA, United States of Amerrica; 4 Public Health Sciences Division, Fred Hutchinson Cancer Center, Seattle, WA, United States of Amerrica; 5 Department of Global Health, University of Washington, Seattle, WA, United States of Amerrica; Janssen Vaccines & Prevention B.V., NETHERLANDS, KINGDOM OF THE

## Abstract

Combination monoclonal broadly neutralizing antibodies (bnAbs) are currently being developed for preventing HIV-1 acquisition. Recent work has focused on predicting in vitro neutralization potency of both individual bnAbs and combination regimens against HIV-1 pseudoviruses using Env sequence features. To predict in vitro combination regimen neutralization potency against a given HIV-1 pseudovirus, previous approaches have applied mathematical models to combine individual-bnAb neutralization and have predicted this combined neutralization value; we call this the combine-then-predict (CP) approach. However, prediction performance for some individual bnAbs has exceeded that for the combination, leading to another possibility: combining the individual-bnAb predicted values and using these to predict combination regimen neutralization; we call this the predict-then-combine (PC) approach. We explore both approaches in both simulated data and data from the Los Alamos National Laboratory’s Compile, Neutralize, and Tally NAb Panels repository. The CP approach is superior to the PC approach when the neutralization outcome of interest is binary (e.g., neutralization susceptibility, defined as inhibitory 80% concentration < 1 μg/mL). For continuous outcomes, the CP approach performs nearly as well as the PC approach when the individual-bnAb prediction algorithms have strong performance, and is superior to the PC approach when the individual-bnAb prediction algorithms have poor performance. This knowledge may be used when building prediction models for novel antibody combinations in the absence of in vitro neutralization data for the antibody combination; this, in turn, will aid in the evaluation and down-selection of these antibody combinations into prevention efficacy trials.

## Introduction

Monoclonal broadly neutralizing antibody (bnAb) regimens for HIV-1 prevention have been researched extensively [1–4]. The Antibody Mediated Prevention (AMP) randomized efficacy trials of VRC01 versus placebo (HVTN 704/HPTN 085 and HVTN 703/HPTN 081, NCT02716675 and NCT02568215, respectively) provided evidence that a bnAb can prevent HIV-1 acquisition [5]. Defining a VRC01-susceptible strain as having 80% inhibitory concentration (IC_80_) <1 μg/mL, the AMP trials showed that estimated prevention efficacy of VRC01 versus placebo for VRC01-susceptible strains was 75.4% [5]. Estimated prevention efficacy for strains with 50% inhibitory concentration (IC_50_) <1 μg/mL was approximately 50% [5]. Combination regimens consisting of multiple bnAbs targeting several HIV-1 Env epitopes have greater *in vitro* neutralization potential than their constituent bnAbs [6–8], and as such are currently being prioritized for HIV-1 prevention [3].

Several methods have been developed for predicting individual [9–17] and both individual and combination [18] in vitro neutralization outcomes from Env sequence data. Many of these algorithms were trained using the Los Alamos National Laboratory’s Compile, Analyze, and Tally NAb Panels (CATNAP) database [19] and yield good prediction performance (small mean squared error or large area under the receiver operating characteristic curve [AUC]) for individual bnAbs, with AUC often much greater than 0.5 [11, 13, 16–18]. One limitation of CATNAP is that while sufficient numbers of pseudoviruses have been measured for neutralization against individual bnAbs, far fewer (and in many cases, zero) have measured neutralization against combination regimens. Additionally, many more pseudoviruses tend to have measurements of IC_50_ than IC_80_, likely due to the higher sensitivity of the former. To maximize the amount of information available when investigating combination regimens, combination neutralization is often defined as a function of the individual neutralization values, and then used as an outcome for training the prediction model [6, 18, 20]. We refer to this as the *combine-then-predict (CP)* approach. Other outcomes, including *multiple susceptibility*—defined as the binary indcator that *k* bnAbs in the regimen have IC_80_ < 1 μg/mL—could be used instead. Each of these models results in a prediction function that takes as input Env sequence data and returns a predicted neutralization outcome. These prediction models may be used to compare bnAb regimens when determining which to pursue in further research [17, 21], since neutralization is associated with prevention efficacy [5, 22].

However, prediction performance for some individual bnAbs has exceeded that for the combination [21]; in some cases, predicting the individual-bnAb neutralization seems to be an easier task than predicting combination neutralization. This suggests a second possible method for predicting combination neutralization outcomes: combining the individual-bnAb predicted values and using these combinations to predict neutralization by the combination regimen. We refer to this as the *predict-then-combine (PC)* approach. In this article, we explore the PC approach and contrast it with the previous CP approach of directly predicting the combined neutralization. We study the performance of both approaches in simulated data and data from CATNAP, aiming to provide clarity on when each approach should be used. We use the term *prediction performance* to refer to a summary of the discrepancy between predictions from an algorithm and observed neutralization outcomes; possibilities include R-squared for continuous outcomes and AUC for binary outcomes, among others.

## Materials and methods

### Predicting neutralization values

Neutralization values can be predicted as a function of Env sequence AA data using standard prediction algorithms for high-dimensional data. It is important to account for the large number of features compared to the often smaller number of sequences with measured neutralization. Hake and Pfeifer [11] compared the lasso [23], random forests [24], and support vector machines [25] (also evaluated in [9]), finding good performance for each method. Neural networks [26] have been used in several studies [10, 14, 17], while Rawi et al. [13] used gradient boosted trees [27]. Finally, Magaret et al. [16] and Williamson et al. [21] used the Super Learner [28], an ensemble method. Each of these prediction approaches has been shown to perform well for predicting HIV neutralization in certain settings.

In our analyses described below (Results), we will focus on the lasso for two reasons. First, it is a commonly-used prediction algorithm that performs well in many settings. More importantly, our goal is to compare prediction performance using the same modeling approach between two methods for combining individual-bnAb neutralization values, rather than building the best possible prediction model for a given outcome.

### Combining individual-bnAb neutralization values

The first method of predicting combination bnAb regimen susceptibility that we consider, which we refer to as pre-prediction combination or the *combine-then-predict* (CP) approach, involves applying a mathematical model to combine measured in vitro inhibitory concentration prior to training a prediction model. We consider two mathematical models defined by Wagh et al. [6] for combining the in vitro neutralization values from *J* constituent bnAbs in a bnAb combination regimen: an additive model,

$$\begin{array}{cc} \text{Additive}\;\text{combination}\;{\text{IC}}_{80}≔ & (\sum_{j=1}^{J}IC_{80,j}^{-1}{)}^{-1}, \end{array}$$ (1)

and a Bliss-Hill model,

$$\begin{array}{cc} \text{Bliss-Hill}\;\text{combination}\;{\text{IC}}_{80}≔ & \underset{c\in [0,1]}{\text{min}}\left|h_{J}\left(c\right)-0.8\right|,\;\text{where}\\ h_{J}\left(c\right)≔ & 1-\prod_{j=1}^{J}\left\{1-h_{j}\left(c,c/J\right)\right\},\\ h_{j}\left(c,c_{j}\right)≔ & \frac{c^{m}}{{\text{IC}}_{50,j}^{m}+c_{j}^{m}},\;\text{and}\\ m≔ & \frac{{\text{log}}_{10}\left(4\right)}{{\text{log}}_{10}\left({\text{IC}}_{80,j}\right)-{\text{log}}_{10}\left({\text{IC}}_{50,j}\right)}. \end{array}$$ (2)

**Fig 1:**
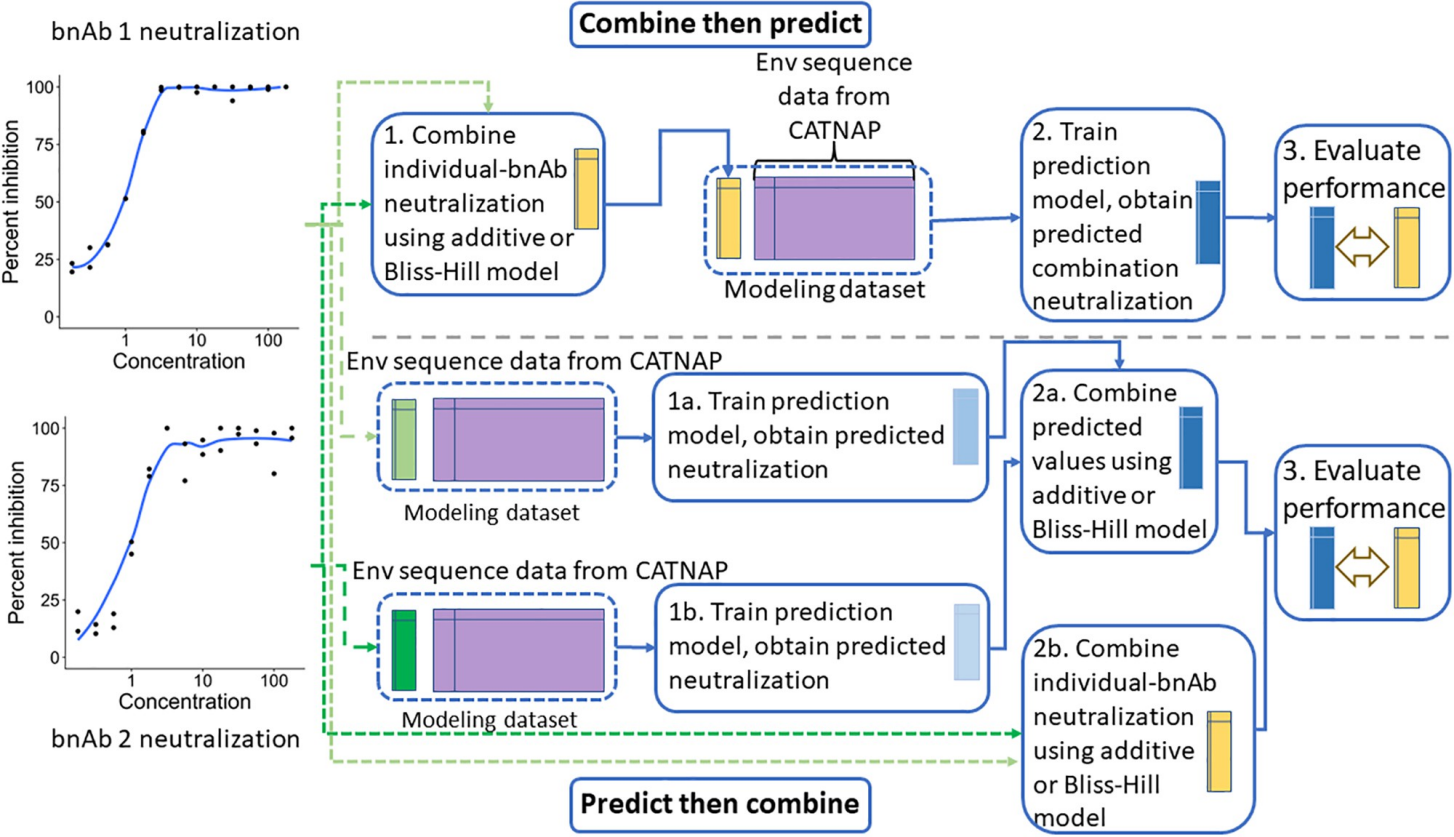
Illustration of combination regimen prediction approaches. An illustration of the combine-then-predict (top) and predict-then-combine (bottom) approaches to predicting combination regimen in vitro neutralization, for a two-bnAb combination regimen.

The Bliss-Hill solution is obtained using the method of Brent [29]. These models have only been validated for combinations where the constituent bnAbs target different HIV-1 Env epitopes [6, 20]. Combination susceptibility based on IC_80_, a binary outcome, is defined as the binary indicator that combination IC_80_ < 1 μg/mL. Combination IC_50_ (the 50% inhibitory concentration) and combination susceptibility based on IC_50_ are defined in a similar manner, with IC_50_ replacing IC_80_ in Eq (1) and *h*_*j*_ in Eq (2). Then, a prediction model can be trained by using the Env features to predict the combination neutralization outcome (either continuous or binary). Any prediction modelling approach can be used: for example, the lasso [23] or random forests [24]. This approach is laid out in the left-hand column of Fig 1.

One could instead perform post-prediction combination, which is the second method of predicting combination bnAb regimen susceptibility that we consider. We will refer to this as the *predict-then-combine* (PC) approach. This method involves first training *J* prediction models using the Env features to predict the continuous neutralization outcome (e.g., IC_80_) of each individual bnAb in the combination regimen. As above, any prediction modelling technique can be used for these individual prediction models. We then obtain predicted neutralization ${\left\{g_{j}\right\}}_{j=1}^{J}$ and use either the additive or Bliss-Hill model to combine the predicted values, using the predictions *g*_*j*_ in place of the measured IC_80,*j*_ or IC_50,*j*_. To predict susceptibility, we check if the combined predicted value is less than 1 μg/mL.

### Creating synthetic datasets

We first consider synthetic datasets where we determine the relationship between Env sequence features and neutralization outcomes. This allows us to evaluate the CP and PC approaches in a controlled setting where we know the true neutralization outcomes. For sample size *n* ∈ {100, 200, …, 1000} and three bnAbs, we generated 1000 Env AA sequence features and IC_80_ values for each bnAb and each of the *n* simulated pseudoviruses. We assumed that conditional on the Env AA sequence features, the IC_80_ values were independent. We ranged the sample size to provide a comprehensive evaluation of the CP and PC approaches for different amounts of data, to see when results between the two approaches separate. In general, prediction performance should improve with increasing sample size. In our simulated setting, only ten Env AA sequence features impact the IC_80_. This setup, where there are a large number of Env AA sequence features but only a small number of highly predictive features, is similar to the setting and results of analyses using CATNAP data [16].

We considered two scenarios: one where there was a weak relationship between these ten Env AA sequence features and IC_80_, and one where there was a strong relationship. The proportion susceptible to each of the three bnAbs is 0.36, 0.29, and 0.42, respectively, in the weak-relationship scenario; and is 0.23, 0.21, and 0.27, respectively, in the strong-relationship scenario. We then obtained true combination IC_80_ using the additive model described above. The proportion susceptible using combination IC_80_ is 0.74 in the weak-relationship scenario and 0.38 in the strong-relationship scenario. We repeated the above data-generating process 2500 times for each sample size.

### Creating CATNAP datasets

We consider several bnAb combination regimens with data available in CATNAP. These regimens consist of those undergoing HIV Vaccine Trials Network (HVTN) or HIV Prevention Trials Network (HPTN) clinical testing as of October 2022 [21] and those with at least 125 pseudoviruses with direct measurement of neutralization by the bnAb combination in CATNAP [18]. The full list of bnAb combination regimens is provided in Table 1. Each combination regimen consists of individual bnAbs targeting different HIV-1 Env epitopes: V1V2, V3, CD4 binding site (CD4bs), fusion peptide, or membrane proximal external region (MPER) [12, 13, 18] (Table 1).

**Table 1:** Broadly neutralizing antibody regimens considered in analyses. Broadly neutralizing antibody (bnAb) combination regimens either with at least 125 sequences in CATNAP with direct measurement of combination neutralization or undergoing HVTN/HPTN clinical testing as of October 2022. The HIV-1 Env epitopes targeted by each bnAb in the combination regimen are listed in order. Combination regimens highlighted in the Results section below are highlighted in bold.

Combination regimen	Epitopes	Number of sequences in CATNAP with IC_80_ data measured against both constituent bnAbs	Number of sequences in CATNAP with IC_80_ data measured against the bnAb combination
BG1 + BG18 + NC37	V1V2, V3,	118	119
PG9 + PGT128	V2, V3	300	125
PG9 + PGT128 + VRC07	V2, V3, CD4bs	300	125
PG9 + VRC07	V2, CD4bs	392	125
PGT128 + VRC07	V3, CD4bs	299	125
**VRC07–523-LS + 10–1074**	CD4bs, V3	400	0
VRC07–523-LS + PGDM1400	CD4bs, V1V2	400	0
VRC07–523-LS + PGT121	CD4bs, V3	400	0
VRC07–523-LS + PGDM1400 + PGT121	CD4bs, V1V2, V3	400	0
VRC07–523-LS + VRC26.25	CD4bs, V1V2	400	0
3BNC117 + PG9	CD4bs, V1V2	400	125
10–1074 + 3BNC117	V3, CD4bs	542	125
10–1074 + 3BNC117 + PG9	V3, CD4bs, V1V2	402	125
**10–1074 + 10E8**	V3, MPER	403	125
10–1074 + 10E8 + 3BNC117	V3, MPER, CD4bs	403	125
10–1074 + PG9	V3, V1V2	402	125
10E8 + 3BNC117	MPER, CD4bs	400	125
10E8 + 3BNC117 + PG9	MPER, CD4bs, V1V2	400	125
10E8 + PG9 + PGT128	MPER, V1V2, V3	300	125
10E8 + PG9 + VRC07	MPER, V1V2, CD4bs	392	125
10E8 + PGT128 + VRC07	MPER, V3, CD4bs	300	125

We began by using the Super LeArner Prediction of NAb Panels (SLAPNAP) tool [18] to create an analysis dataset for each unique bnAb in Table 1 consisting of individual-bnAb IC_50_ and IC_80_ values, along with Env gp160 sequence features. We also obtained datasets of directly measured combination neutralization for those bnAb regimens in Table 1 with such information (Table 1 column 3 not equal to zero). The features for each pseudovirus consist of geographic information (binary indicator variables describing the geographic region of origin), viral geometry variables (length, number of sequons, and number of cysteines in the Env, gp120, V2, V3, and V5 regions), and amino acid sequence variables (binary indicators of residues containing amino acids, frameshifts, gaps, stops, or sequons at each HXB2-referenced site in gp160). Each dataset had approximately 6000 columns.

We next merged the individual-bnAb neutralization values and directly-observed combination neutralization values (if available) into a single dataset for each bnAb regimen in Table 1. We computed model-predicted combination neutralization using both the additive and Bliss-Hill models described above. The statistical learning approaches described below aimed to predict the following neutralization outcomes for each individual bnAb in the regimen: (1) the continuous log_10_ IC_50_; (2) the continuous log_10_ IC_80_; (3) the dichotomous outcome IC_50_ susceptibility, defined as IC_50_ < 1 μg/mL; and (4) the dichotomous outcome IC_80_ susceptibility, defined as IC_80_ < 1 μg/mL. We also aimed to predict the following outcomes for each combination regimen and both the additive and Bliss-Hill combination models: (5) the continuous log_10_ combination IC_50_; (6) the continuous log_10_ combination IC_80_; (7) the dichotomous outcome combination IC_50_ susceptibility, defined as combination IC_50_ < 1 μg/mL; and (8) the dichotomous outcome combination IC_80_ susceptibility, defined as combination IC_80_ < 1 μg/mL. For combination regimens with directly-observed combination neutralization, we further aimed to predict outcomes (1) through (4) but using the directly-observed combination neutralization values.

### Obtaining predictions and assessing prediction performance

We took the same approach to obtaining predictions and assessing prediction performance in both the synthetic and CATNAP data settings. For each given dataset, we used the lasso [23] to (i) predict the individual-bnAb neutralization (both continuous and binary); (ii) predict the combination neutralization directly (both continuous and binary), i.e., use the CP approach; and (iii) combine the continuous-valued predictions from (i) using the additive model, i.e., use the PC approach. We used 10-fold cross-validation to obtain the optimal lasso tuning parameter in all cases. We then assessed prediction performance using an additional layer of 10-fold cross-validation, where the lasso was trained on nine-tenths of the data and prediction performance was estimated on the remaining tenth. We evaluated prediction performance using cross-validated (CV) R-squared for continuous outcomes and cross-validated area under the receiver operating characteristic curve (CV AUC) for binary outcomes. We also obtained a 95% confidence interval for the prediction performance [30, 31].

In the synthetic setting, we know the true combination neutralization values. Prediction performance is evaluated using these observed values and the predictions from either the CP or PC approach. We computed the average point estimate, empirical standard error of the point estimates, and average confidence interval width after applying the prediction performance estimation procedure described above to each of the 2500 datasets.

In the CATNAP setting, we only know the true combination neutralization values for those bnAb regimens in Table 1 with a nonzero number of sequences with IC_80_ data measured against the bnAb combination. We evaluated individual-bnAb prediction performance as described above. Performance for predicting combination regimen neutralization was measured by either combining the individual-bnAb predictions or by directly predicting the combination neutralization. We considered both the additive and Bliss-Hill models described above for combining neutralization values. For a given combination regimen of interest, to evaluate both the CP and PC approaches fairly, we held out pseudoviruses with observed neutralization for the combination regimen as a test set.

### Software and compiled datasets

The procedures described above (and the results described below) can be reproduced using code available on GitHub at https://github.com/bdwilliamson/hiv_neutralization_susceptibility_supplementary and at Zenodo (https://zenodo.org/doi/10.5281/zenodo.10372961). In addition to code, these repositories host compiled datasets following the procedure described in Methods (Creating CATNAP datasets). We used the latest compiled version of SLAPNAP [18] to compile CATNAP datasets, using Docker [32] version 24.0.0. All analyses were performed using R [33]; the simulations were performed in version 4.3.1, while the data analyses were performed in version 4.2.2. Specific R packages and versions used are: here version 1.0.1 [34], readr version 2.1.5 [35], janitor version 2.2.0 [36], glmnet version 4.1.8 [37, 38], data.table version 1.15.4 [39], dplyr version 1.1.4 [40], tidyr version 1.3.1 [41], parallel version 4.4.0 [33], foreach version 1.5.2 [42], doParallel version 1.0.17 [43], cvAUC version 1.1.4 [44], vimp version 2.3.3 [30, 45], ggplot2 version 3.5.1 [46], cowplot version 1.1.3 [47], and gridExtra version 2.3 [48].

## Results

### Performance of combine-then-predict and predict-then-combine on synthetic data

**Fig 2:**
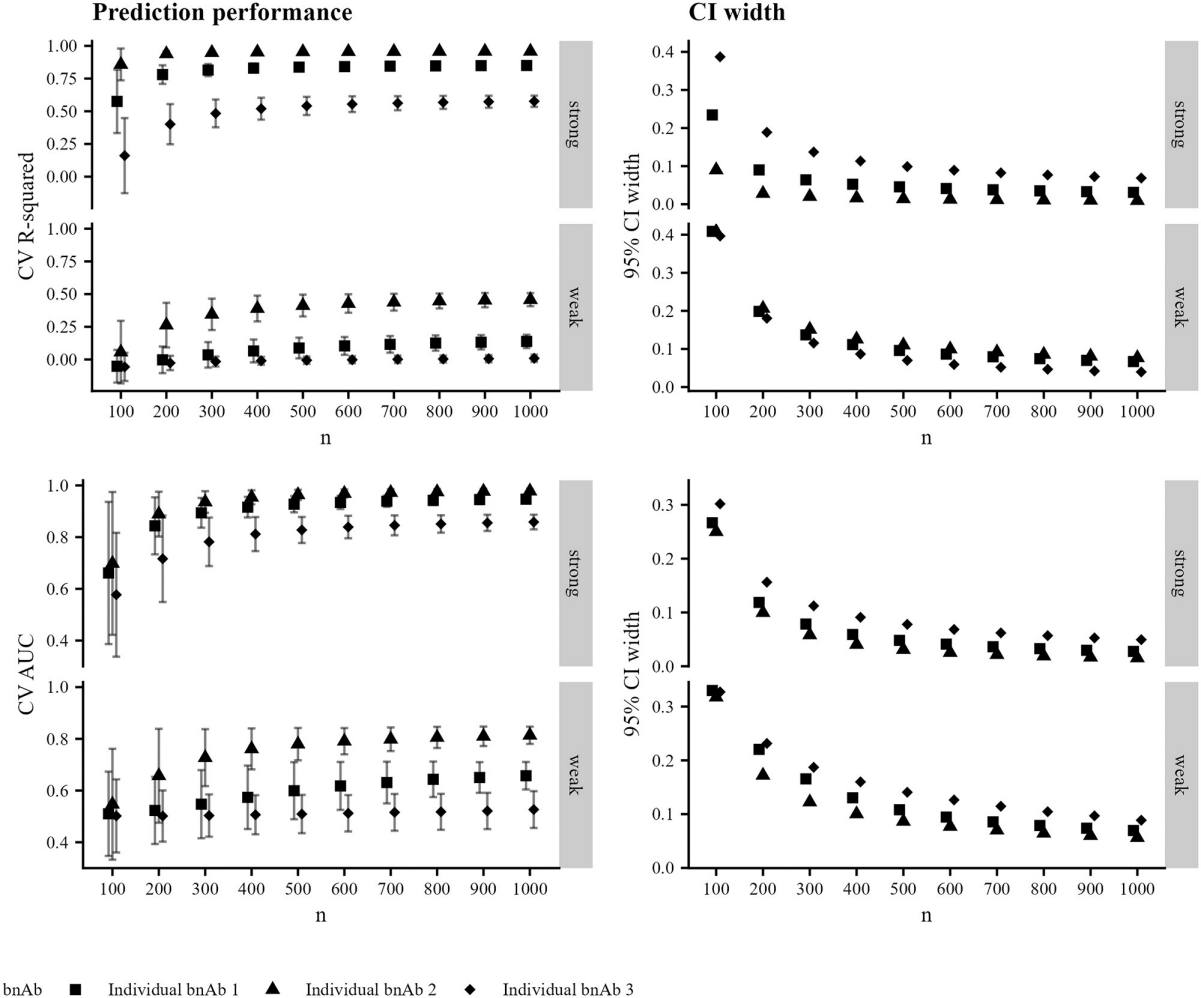
Prediction performance for individual bnAbs in synthetic experiments. Prediction performance (left-hand column) and 95% confidence interval width (right-hand column) versus sample size for predicting IC_80_ (top row) and IC_80_ < 1 μg/mL for each of three individual bnAbs averaged over 2500 simulated datasets. Prediction performance is evaluated against the observed IC_80_ values for the given bnAb. Panels within rows denote a strong or weak relationship between the Env AA predictors and the outcome. Shapes denote the individual bnAbs, and Monte-Carlo error is displayed in error bars.

**Fig 3:**
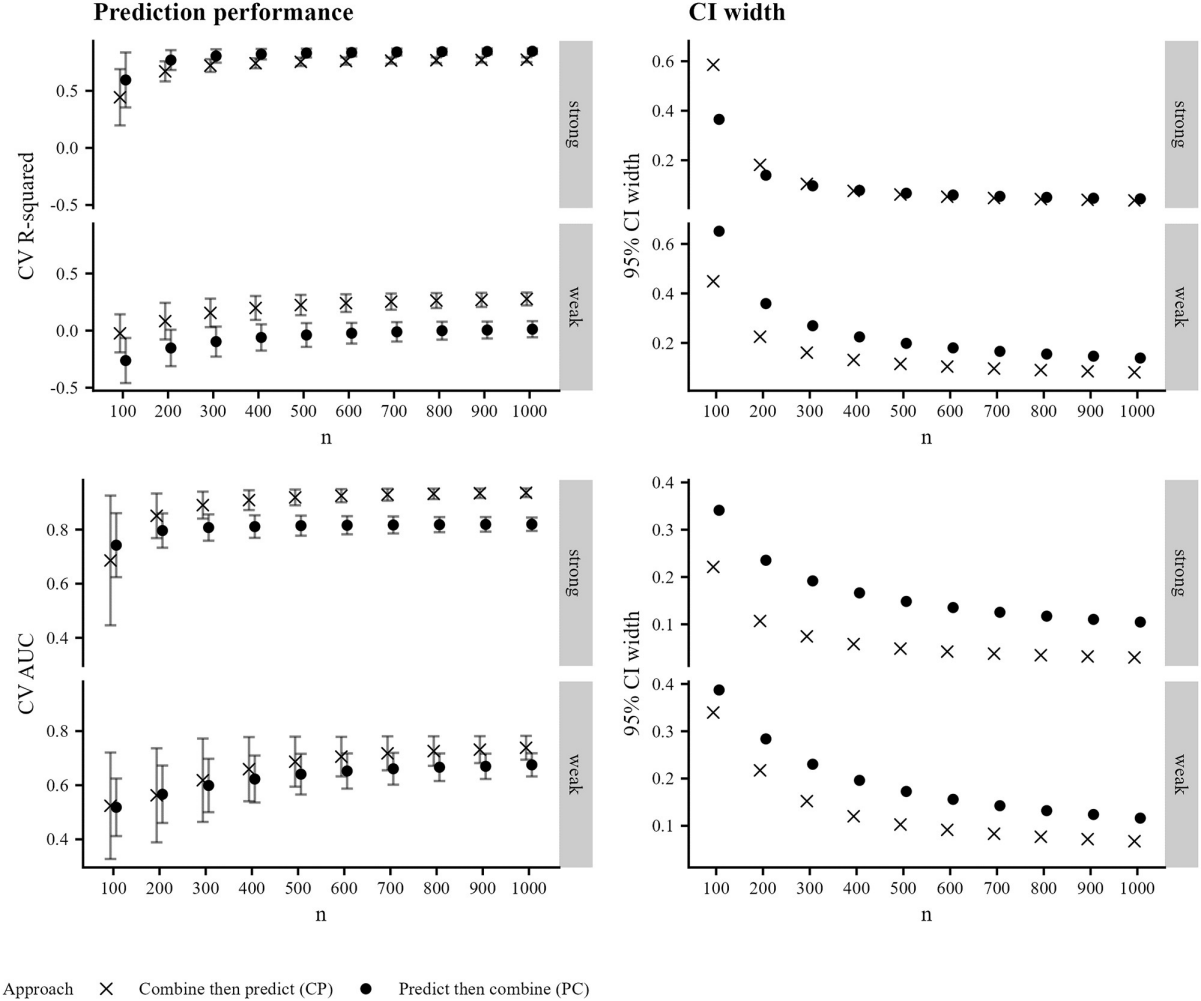
Prediction performance for combination regimens in synthetic experiments. Prediction performance (left-hand column) and 95% confidence interval width (right-hand column) versus sample size for predicting combination IC_80_ (top row) and combination IC_80_ < 1 μg/mL using both the CP and PC approaches, averaged over 2500 simulated datasets. Prediction performance is evaluated against the observed combination IC_80_. Panels within rows denote a strong or weak relationship between the Env AA predictors and the outcome. Shapes denote the approach, and Monte-Carlo error is displayed in error bars.

We display the results for the simulated data described in Methods (Creating synthetic datasets) in Figs 2 and 3. In Fig 2 (left-hand column), we see that the prediction performance varies across individual bnAbs in the combination regimen, with the second bnAb having uniformly highest prediction performance; the third bnAb having uniformly lowest prediction performance; and the first bnAb having prediction performance between the other two. Prediction performance is degraded in the weak-relationship case, which is expected. Confidence interval width decreases with increasing sample size for both outcomes, as expected (right-hand column of Fig 2), though width tends to be higher in the weak-relationship case than in the strong-relationship case. In Fig 3 (left-hand column), we see that prediction performance is similar across the two methods of predicting combination neutralization for the continuous outcome (nearly within Monte-Carlo error) in the strong-relationship case, but is uniformly higher when using the CP approach in the weak-relationship case, corresponding to a case where CV R-squared for all individual bnAbs was less than 0.5. Training prediction models using the CP approach results in higher prediction performance for susceptibility regardless of the strength of relationship between predictors and outcome. Confidence interval width is uniformly smaller for the CP method (Fig 3 right-hand column).

### Performance on bnAb combinations in CATNAP

We further explored the distinction between the CP and PC approaches by obtaining prediction performance for the bnAb combination regimens in Table 1. We followed the approach described in Methods (Creating CATNAP datasets) to define the dataset for each bnAb regimen.

For the bnAb regimens with direct lab-measured neutralization for the combination regimen (third column of Table 1), we further assessed the performance of both approaches for predicting these neutralization values. Below, we highlight the results for two bnAb regimens, VRC07–523-LS + 10–1074 (chosen because it is a regimen in clinical testing) and 10–1074 + 10E8 (chosen because it is a regimen with direct measurement of neutralization for the regimen). Since predicting the directly-measured neutralization against a combination regimen is an important goal, in cases where this direct measurement exists we evaluate prediction performance for this outcome as well as the model-combined outcome.

The proportion of pseudoviruses in CATNAP susceptible (measured using IC_80_, defined as IC_80_ < 1 μg/mL) to VRC07–523-LS, 10–1074, and 10E8 are 78.75%, 48.89%, and 21.34%, respectively. The proportion estimated to be sensitive to VRC07–523-LS + 10–1074 is 89.5% and 93.5% for the additive and Bliss-Hill models, respectively; estimated sensitivity is computed using the model based on the individual-bnAb neutralization data in CATNAP, as described in Methods (Combining individual-bnAb neutralization values). The proportion estimated to be sensitive to 10–1074 + 10E8 is 59.65% and 79.7% for the additive and Bliss-Hill models, respectively, while the true proportion susceptible is 64.8%. This true proportion is based on a direct measurement of IC_50_ for the combination regimen in CATNAP (Table 1; see Methods (Creating CATNAP datasets)). Based on the number of pseudoviruses, the number of susceptible viruses is comparable to *n* ∈ {600, …, 1000} in the simulations above. Results for the remaining bnAb regimens from Table 1 can be found in S1 Appendix.

**Fig 4:**
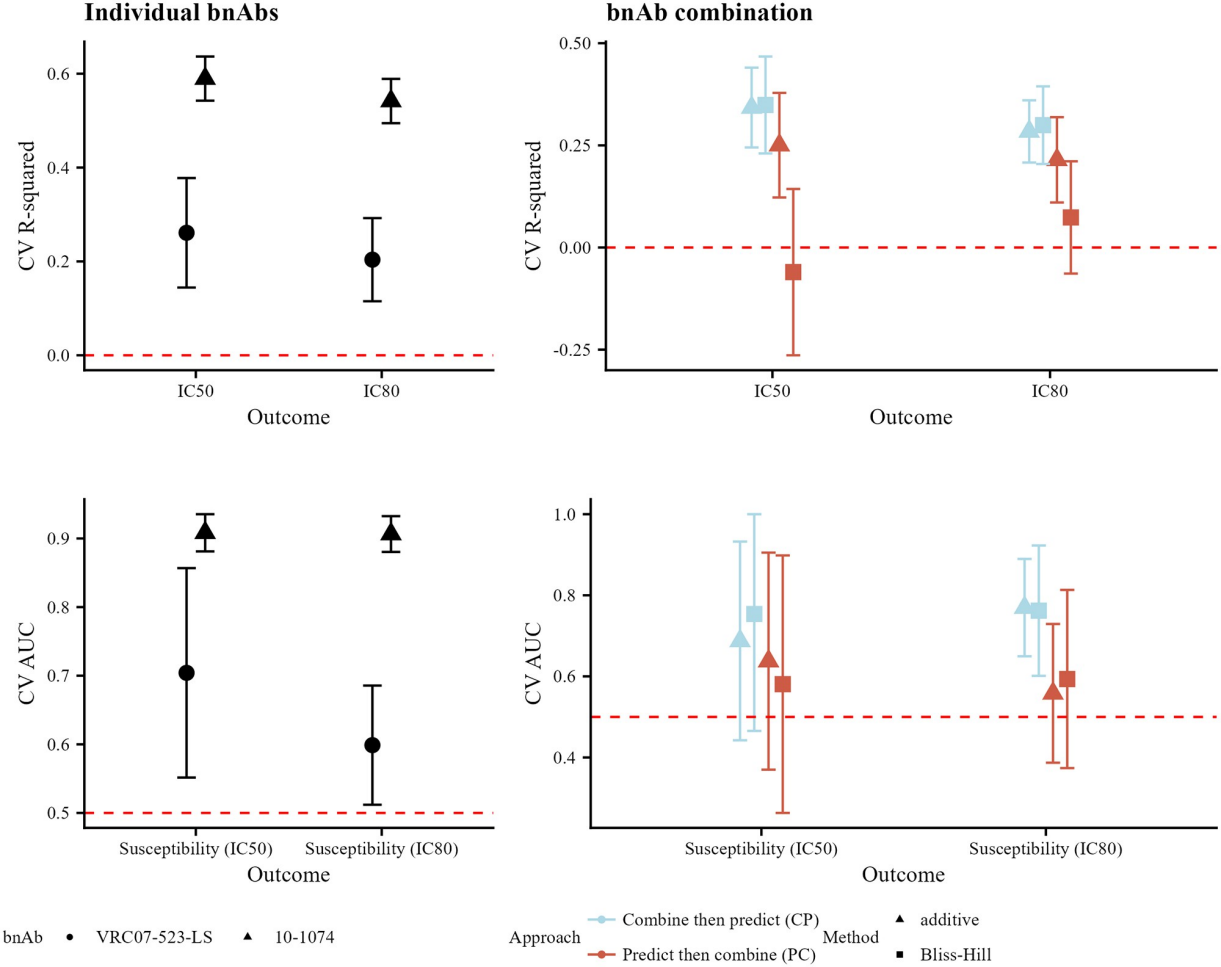
Prediction performance in CATNAP for VRC07–523-LS + 10–1074. Prediction performance for continuous (top row, CV R-squared) and binary (bottom row, CV AUC) neutralization outcomes for individual bnAbs (left-hand column) and the combination (right-hand column) VRC07–523-LS + 10–1074. For individual bnAbs, prediction performance is evaluated against the observed IC_50_ or IC_80_ values for the given bnAb; shapes denote the bnAb. For combination bnAbs, prediction performance is evaluated against the calculated combination IC_50_ or IC_80_ values based on the observed bnAb-specific values using the additive or Bliss-Hill method; shapes denote the combination method (additive or Bliss-Hill) and color denotes the approach (CP or PC). Error bars reflect 95% confidence intervals.

We display the results for predicting neutralization susceptibility to VRC07–523-LS + 10–1074 in Fig 4. In the left-hand column, we see that prediction performance is higher for 10–1074 for both continuous and binary neutralization outcomes. In the right-hand column, we see that for both continuous and binary outcomes, the CP approach leads to better prediction performance (the CV R-squared ranges from 0.07 to 0.41 lower for PC compared to CP; the CV AUC ranges from 0.05 to 0.21 lower). This improved performance of the CP approach is particularly striking for the Bliss-Hill model in the continuous-outcome setting. This combination regimen does not have direct measurements of neutralization by the combination regimen in CATNAP. As in the simulations, prediction performance for the combination tends to be less than the best individual-bnAb prediction performance. When using the CP approach, prediction performance for the combination tends to be greater than the worst individual-bnAb prediction performance.

**Fig 5:**
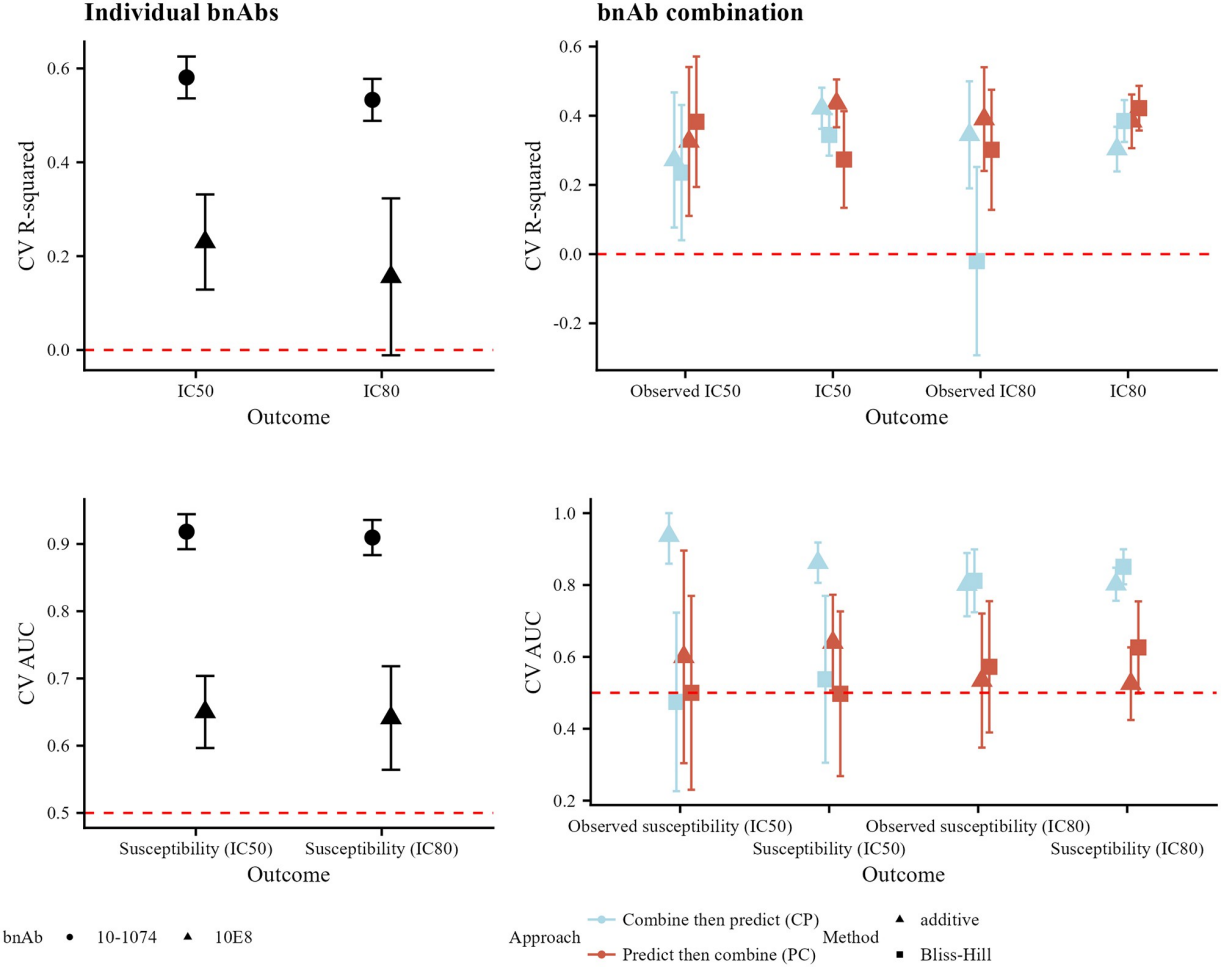
Prediction performance in CATNAP for 10–1074 + 10E8. Prediction performance for continuous (top row, CV R-squared) and binary (bottom row, CV AUC) neutralization outcomes for individual bnAbs (left-hand column) and the combination (right-hand column) 10–1074 + 10E8. For individual bnAbs, prediction performance is evaluated against the observed IC_50_ or IC_80_ values for the given bnAb; shapes denote the bnAb. For combination bnAbs, prediction performance is evaluated against both the observed IC_50_ or IC_80_ values based on the bnAb regimen (denoted by the prefix “observed”) and the calculated combination IC_50_ or IC_80_ values based on the observed bnAb-specific values using the additive or Bliss-Hill method; shapes denote the combination method (additive or Bliss-Hill) and color denotes the approach (CP or PC). Error bars reflect 95% confidence intervals.

We display the results for predicting neutralization susceptibility to 10–1074 + 10E8 in Fig 5. In the left-hand column, we see that prediction performance is again higher for 10–1074 for both continuous and binary neutralization outcomes. In the right-hand column, we see that prediction performance for continuous outcomes is similar between the CP and PC approaches (CV R-squared ranges from 0.07 lower for PC compared to CP [Bliss-Hill model, IC_50_] to 0.32 higher [Bliss-Hill model, directly-observed combination IC_80_]); for binary outcomes, performance is better when using the CP approach (CV AUC ranges from 0.03 higher for PC compared to CP [Bliss-Hill model, directly-observed IC_50_ susceptibility] to 0.34 lower [additive model, directly-observed IC_50_ susceptibility]). This antibody combination does have direct measurements of neutralization by the combination regimen in CATNAP, and the results follow a similar pattern to the combination neutralization results. These results are similar to the results from the simulations.

Results for the remaining bnAb regimens from Table 1 followed similar patterns (S1 Appendix). We first compared the performance of the Bliss-Hill and additive models across all combination regimens with directly-observed combination neutralization. For IC_50_, the mean difference in CV R-squared was −0.05 with interquartile range (IQR) (−0.08, 0.17) and the mean difference in CV AUC was −0.10 with IQR (−0.13, 0); for IC_80_, the mean difference in CV R-squared was −0.02 with IQR (−0.22, 0.19) and the mean difference in CV AUC was −0.01 with IQR (−0.04, 0.04). We observed that prediction performance for binary neutralization outcomes (e.g., IC_80_ < 1 μg/mL) was often better when using the CP approach than when using the PC approach [IQR of the difference in CV AUC = (−0.13, 0.05) when comparing PC to CP across all combination regimens]. The CP approach is not uniformly better than the PC approach for continuous outcomes, but is often at least as good as the PC approach [IQR of the difference in CV R-squared = (−0.46, −0.07) when comparing PC to CP across all combination regimens]. The results reflect what we saw in the simulations: when continuous-outcome prediction performance for the individual bnAbs is higher than a CV R-squared of 0.5 (e.g., for 10–1074 + 10E8 above, or 10–1074 + PG9, S14 Fig in S1 Appendix) then prediction performance is similar using either the CP or PC approach. For many combinations, however, the CP approach results in better prediction performance than the PC approach.

## Discussion

When predicting virus neutralization susceptibility to a combination bnAb regimen, a key consideration is whether to directly predict the combination neutralization susceptibility (often combined using the individual-bnAb neutralization values due to a lack of data on the combination regimen), which we call the combine-then-predict (CP) approach, or to combine the predictions of individual-bnAb neutralization, which we call the predict-then-combine (PC) approach. In both simulated experiments and our data analysis, we found that prediction performance for binary neutralization outcomes (e.g., IC_80_ < 1 μg/mL) was better using the CP approach compared to the PC approach. Results for continuous outcomes were mixed: in the simulations and the data analysis, we observed that in small samples, prediction performance for combination neutralization was similar between the two approaches. In larger samples (in the simulations), the PC approach seems to result in slightly better prediction performance, when individual-model prediction performance is high. This knowledge may be used when building prediction models for novel antibody combinations in the absence of in vitro neutralization data for the antibody combination; this, in turn, will aid in the evaluation and down-selection of these antibody combinations into prevention efficacy trials.

It is intuitive that increased individual-model prediction performance leads to increased PC performance, since as we noted above prediction errors may be compounded in this approach. The CP approach is similar to averaging IC_80_ values over technical replicates, which generally improves the signal-to-noise ratio (SNR). This is similar to the phenomenon observed in Huang et al. [49], where the authors observed that averaging several immune response endpoints prior to ranking vaccine regimens resulted in better selection of vaccine regimens than ranking the immune response endpoints and then averaging the ranks. The method of Follmann et al. [50] has also been used to compare multiple immune response biomarkers [51], and considers the SNR for each immune response. Applying this method to the simulated examples yields that the SNR for the combination susceptibility is always higher than the SNR for the individual-bnAb susceptibilities, while the SNR for the combination IC_80_ is only higher than the SNR for the individual-bnAb IC_80_ values in the case where the individual-bnAb prediction performance is high; the PC approach performed better than the CP approach in this case. In cases with high SNR for the individual bnAb readouts, denoising through averaging (i.e., using the CP approach) may be less important. This suggests that the SNR can be used as a tool for evaluating whether the CP or PC approach may perform better in practice. However, in all cases, the CP approach led to narrower confidence intervals for prediction performance.

All comparisons between the combine-then-predict and predict-then-combine approaches appear to be more pronounced for the Bliss-Hill model than for the additive model. In particular, the prediction performance results differed between CP and PC more when using the Bliss-Hill model than when using the additive model. This may be due in part to the fact that the Bliss-Hill model uses both IC_50_ and IC_80_, so any prediction error in the individual-bnAb models is compounded; the additive model relies only on predictions of either IC_50_ or IC_80_. While these mathematical models have only been validated for combination regimens where the bnAbs target different Env epitopes, there may be utility in considering regimens that target overlapping epitopes [52].

The CP approach has been taken in prior work where the focus has been developing prediction models for combination neutralization [17, 18, 21]. The results presented here provide evidence that these prior findings are robust, and suggest that the PC approach would not have resulted in increased prediction performance. Thus, future work for predicting combination neutralization should focus on the CP approach, even if prediction performance for individual bnAbs is high.

This study has several limitations. The first is that we only used a single prediction method, the lasso, for our analysis of the CATNAP data. Several groups of authors have observed that either an ensemble of multiple prediction methods [18] or more flexible prediction methods [11, 13, 17] can improve prediction performance using data from CATNAP. While we could certainly observe increased prediction performance by using a more flexible prediction method, comparisons of the CP and PC approaches within a bnAb regimen are nonetheless valid since the predicted values from each approach are evaluated in the same manner regardless of the prediction model used to obtain these values. Additionally, as we saw in the simulations, a CV R-squared greater than approximately 0.5 for all bnAbs in a regimen is necessary for the PC approach to outperform the CP approach; in previous work using an ensemble prediction method, we observed very few CV R-squared values greater than 0.5 across a large number of individual bnAbs [18]. Second, all of the predictions in this work are based only on the HIV-1 Env sequence and do not account for the bnAb variable region amino acid sequence, which has been observed to increase prediction performance [17]. Third, in the simulations, the lasso model provides an unbiased estimator of the true data-generating model, so it is unlikely that prediction performance could be improved. A related limitation is that there is relatively little neutralization data in CATNAP on combination regimens. This limits our ability to either develop prediction models using the neutralization outcomes based on the combination regimen (here, we held these viruses out as an independent test set) or to obtain precise estimates of prediction performance. Fourth, all of the predictions in this work focuses primarily on parental form bnAbs. Common mutations such as methionine-to-leucine (L) and asparagine-to-serine (S) substitutions in the Fc region of bnAbs are expected to improve stability, half-life, and ease of manufacturing, but not neutralization. Therefore, we do not expect our results would change for such LS-formulated bnAbs and combinations. Finally, when direct neutralization data exist in CATNAP for a combination regimen, one could use a meta-learner or ensemble approach [53] to predict the combination neutralization, by predicting individual neutralization for each antibody and fitting a second-stage prediction model with the individual-antibody predictions as features. This approach, while not broadly applicable due to limited combination neutralization data in CATNAP, should be pursued in future research.

## Conclusion

We investigated two approaches to predicting HIV-1 neutralization susceptibility to combination broadly neutralizing antibody (bnAb) regimens in cases where direct measurement of susceptibility is unavailable. We found that in most cases, combining neutralization values from the individual bnAbs and predicting this value resulted in better prediction performance than predicting the individual bnAb values and combining the predicted results.

## Supporting information

S1 Appendix Supplementary materials. Links to the GitHub repository with code for replicating our results, and additional results from the other bnAb combinations in CATNAP (Table 1) that were not presented in the main manuscript.
(PDF)
