# Supplementary material for: Predicting neutralization susceptibility to combination HIV-1 monoclonal broadly neutralizing antibody regimens
Source: PLoS One. 2024 Sep 6;19(9):e0310042. doi: 10.1371/journal.pone.0310042 (PMC11379218; doi:10.1371/journal.pone.0310042)
Supplement: S1 Appendix — Links to the GitHub repository with code for replicating our results, and additional results from the other bnAb combinations in CATNAP (Table 1) that were not presented in the main manuscript. (PDF) [file pone.0310042.s001.pdf]

Supplementary Materials for "Predicting neutralization susceptibility to combination HIV-1 monoclonal broadly neutralizing antibody regimens"

Brian D. Williamson[1,2,3,*], Liana Wu[2], Yunda Huang[2,4] and Peter B. Gilbert[2,3]

[1]Biostatistics Division, Kaiser Permanente Washington Health Research Institute
[2]Vaccine and Infectious Disease Division, Fred Hutchinson Cancer Research Center
[3]Department of Biostatistics, University of Washington
[4]Department of Global Health, University of Washington
[*]brian.d.williamson@kp.org

August 23, 2024

1 Replicating all results

All results can be replicated using code available on GitHub at `https://github.com/bdwilliamson/hiv_neutralization_susceptibility_supplementary`. The `README` files in each directory include instructions for how to replicate the results.

2 Additional results from bnAb combinations in CATNAP

In the main manuscript, we presented results for the bnAb combinations VRC07-523-LS + 10-1074 and 10-1074 + 10E8. Here, we present the results from the remaining bnAb combinations from Table 1 in the main manuscript. In Table 1, we describe the proportions susceptible to each bnAb within the combination and the overall combination.

As a reminder, for each bnAb regimen, we followed the same procedure as in the simulations in the main manuscript: we estimated prediction performance for both continuous and binary outcomes (both based on IC_{50} and IC_{80}) using ten-fold cross-validation, with ten-fold cross-validation to select the lasso tuning parameters. As before, we evaluated both individual-bnAb prediction performance and performance for predicting combination regimen neutralization by either combining the individual-bnAb predictions or by directly predicting the combination neutralization. In this case, we considered both the additive and Bliss-Hill models for combining neutralization values. For the bnAb regimens with lab-measured neutralization for the combination regimen, we further assessed the performance of both approaches for predicting these neutralization values.

We display the results for predicting neutralization susceptibility in order of Table 1 (except for VRC07-523-LS + 10-1074 and 10-1074 + 10E8, which we omit because the results are presented in the main manuscript). We display the full prediction results in Figures 1–18. As in the main manuscript, we see that prediction performance for binary neutralization outcomes (e.g., $IC_{80} < 1$ µg/mL) is better when combining the individual-bnAb neutralization values and then predicting this combined value than when combining individual-bnAb predictions. Results for continuous outcomes are mixed. However, the results reflect what we saw in the simulations: when continuous-outcome prediction performance for the individual bnAbs is higher than 0.5 (e.g., for 10-1074 + 10E8 in the main manuscript, or 10-1074 + PG9, Figure 14) then prediction performance can be similar for continuous combination outcomes when using either pre- or post-prediction combination. For many combinations, however, combining before prediction results in better prediction performance.

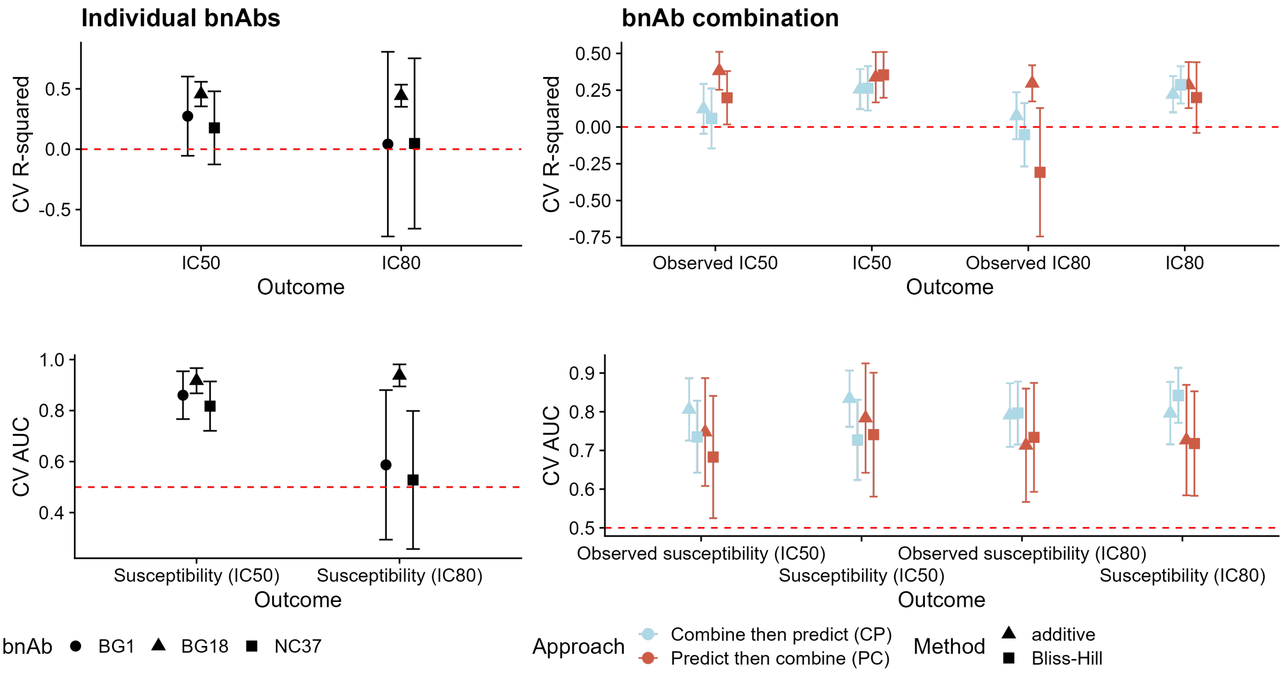

Figure 1: Prediction performance for continuous (top row, CV R-squared) and binary (bottom row, CV AUC) neutralization outcomes for individual bnAbs (left-hand column) and the combination (right-hand column) BG1 + BG18 + NC37. For individual bnAbs, prediction performance is evaluated against the observed IC_{50} or IC_{80} values for the given bnAb; shapes denote the bnAb. For combination bnAbs, prediction performance is evaluated against both the observed IC_{50} or IC_{80} values based on the bnAb regimen (denoted by the prefix "observed") and the calculated combination IC_{50} or IC_{80} values based on the observed bnAb-specific values using the additive or Bliss-Hill method; shapes denote the combination method (additive or Bliss-Hill) and color denotes the approach (CP or PC). Error bars reflect 95% confidence intervals.

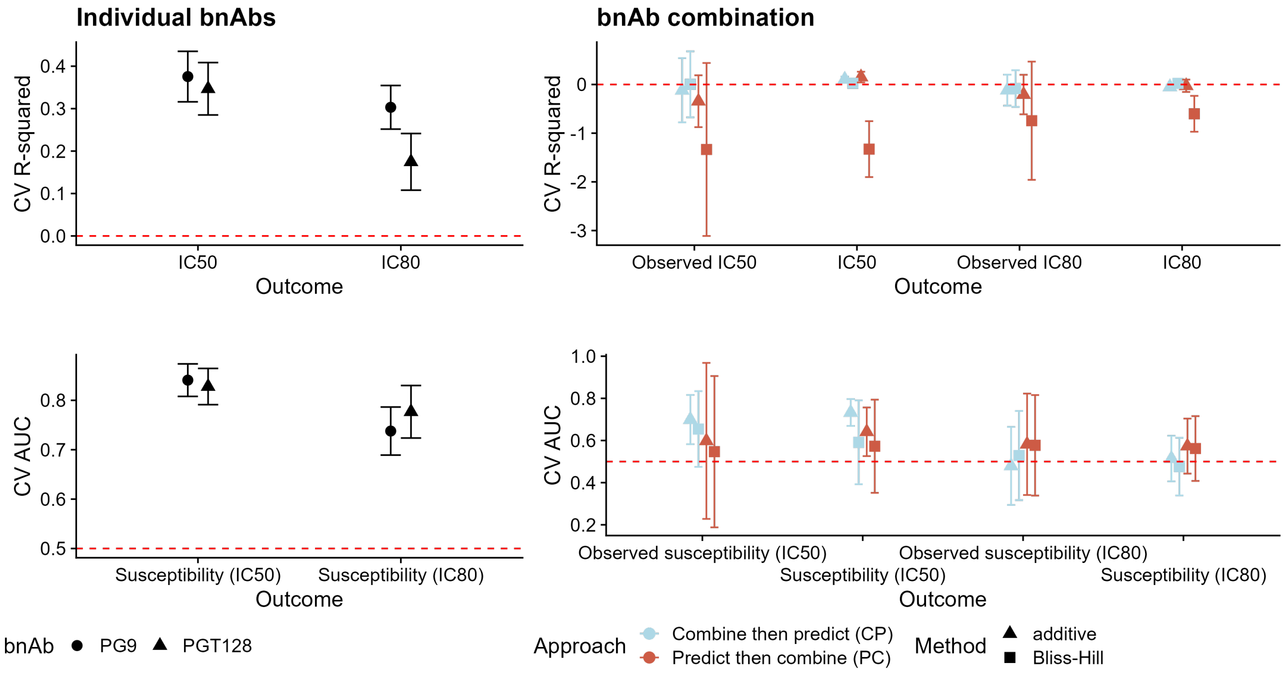

Figure 2: Prediction performance for continuous (top row, CV R-squared) and binary (bottom row, CV AUC) neutralization outcomes for individual bnAbs (left-hand column) and the combination (right-hand column) PG9 + PGT128. For individual bnAbs, prediction performance is evaluated against the observed IC_{50} or IC_{80} values for the given bnAb; shapes denote the bnAb. For combination bnAbs, prediction performance is evaluated against both the observed IC_{50} or IC_{80} values based on the bnAb regimen (denoted by the prefix "observed") and the calculated combination IC_{50} or IC_{80} values based on the observed bnAb-specific values using the additive or Bliss-Hill method; shapes denote the combination method (additive or Bliss-Hill) and color denotes the approach (CP or PC). Error bars reflect 95% confidence intervals.

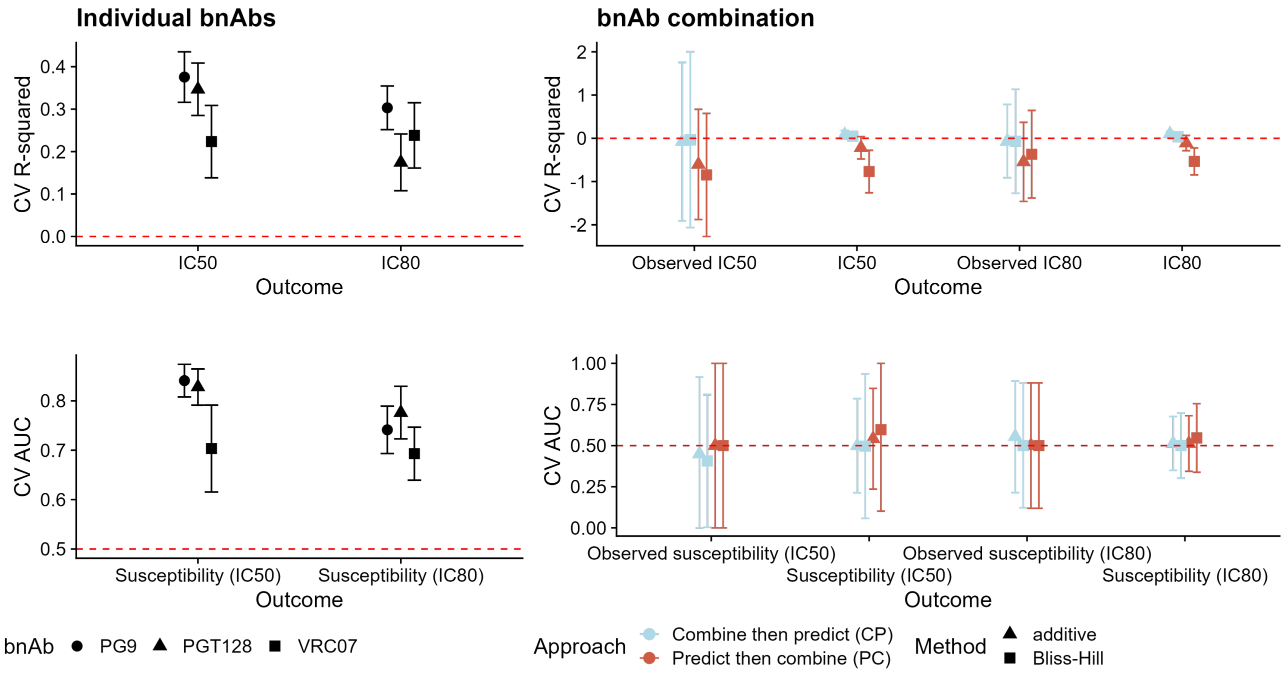

Figure 3: Prediction performance for continuous (top row, CV R-squared) and binary (bottom row, CV AUC) neutralization outcomes for individual bnAbs (left-hand column) and the combination (right-hand column) PG9 + PGT128 + VRC07. For individual bnAbs, prediction performance is evaluated against the observed IC_{50} or IC_{80} values for the given bnAb; shapes denote the bnAb. For combination bnAbs, prediction performance is evaluated against both the observed IC_{50} or IC_{80} values based on the bnAb regimen (denoted by the prefix "observed") and the calculated combination IC_{50} or IC_{80} values based on the observed bnAb-specific values using the additive or Bliss-Hill method; shapes denote the combination method (additive or Bliss-Hill) and color denotes the approach (CP or PC). Error bars reflect 95% confidence intervals.

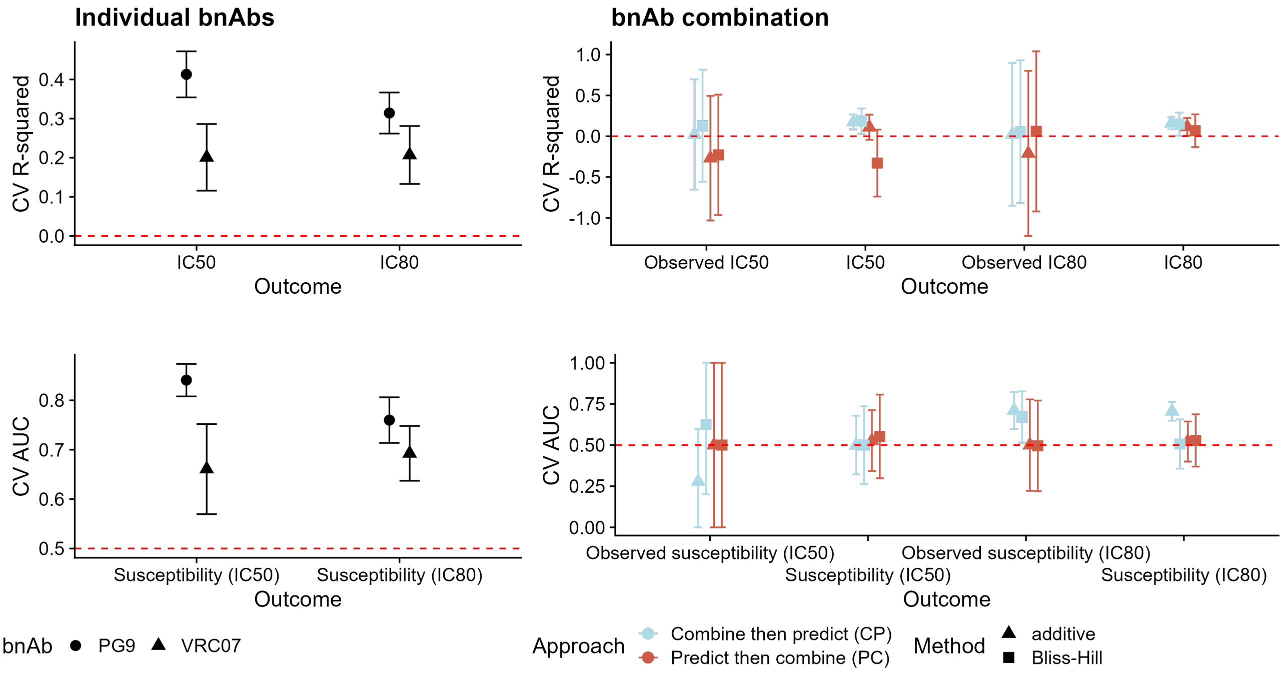

Figure 4: Prediction performance for continuous (top row, CV R-squared) and binary (bottom row, CV AUC) neutralization outcomes for individual bnAbs (left-hand column) and the combination (right-hand column) PG9 + VRC07. For individual bnAbs, prediction performance is evaluated against the observed IC_{50} or IC_{80} values for the given bnAb; shapes denote the bnAb. For combination bnAbs, prediction performance is evaluated against both the observed IC_{50} or IC_{80} values based on the bnAb regimen (denoted by the prefix "observed") and the calculated combination IC_{50} or IC_{80} values based on the observed bnAb-specific values using the additive or Bliss-Hill method; shapes denote the combination method (additive or Bliss-Hill) and color denotes the approach (CP or PC). Error bars reflect 95% confidence intervals.

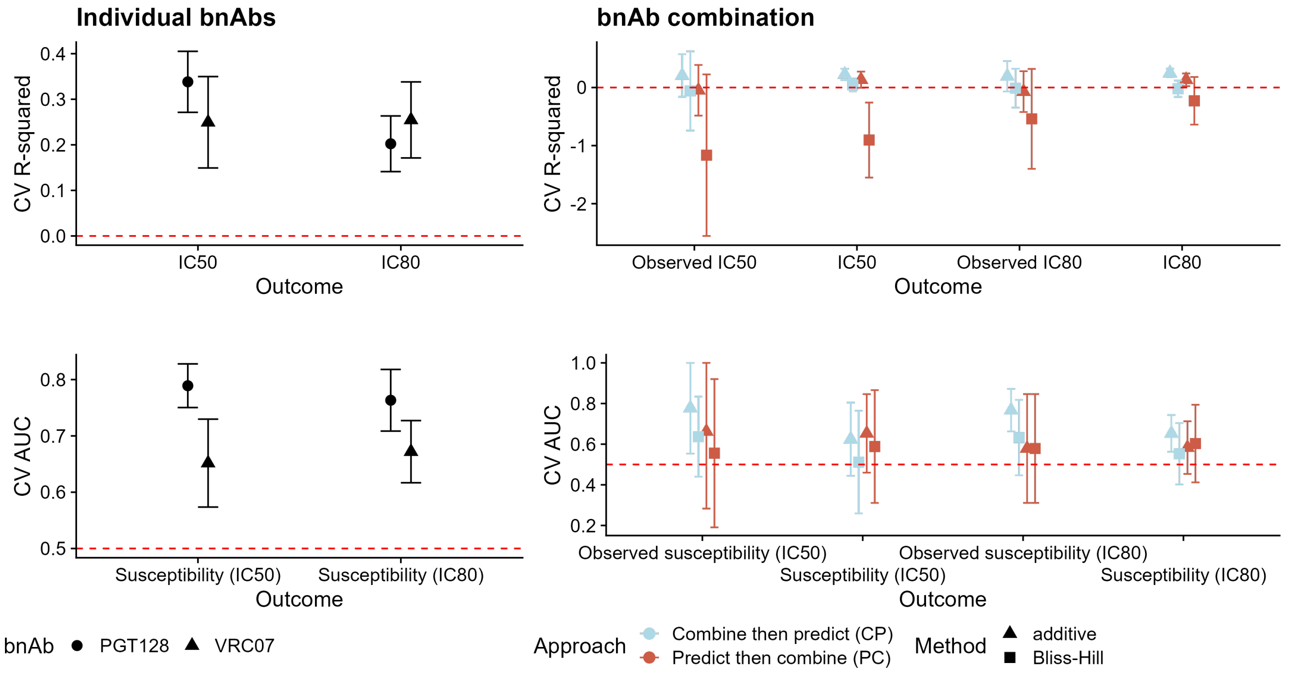

Figure 5: Prediction performance for continuous (top row, CV R-squared) and binary (bottom row, CV AUC) neutralization outcomes for individual bnAbs (left-hand column) and the combination (right-hand column) PGT128 + VRC07. For individual bnAbs, prediction performance is evaluated against the observed IC_{50} or IC_{80} values for the given bnAb; shapes denote the bnAb. For combination bnAbs, prediction performance is evaluated against both the observed IC_{50} or IC_{80} values based on the bnAb regimen (denoted by the prefix "observed") and the calculated combination IC_{50} or IC_{80} values based on the observed bnAb-specific values using the additive or Bliss-Hill method; shapes denote the combination method (additive or Bliss-Hill) and color denotes the approach (CP or PC). Error bars reflect 95% confidence intervals.

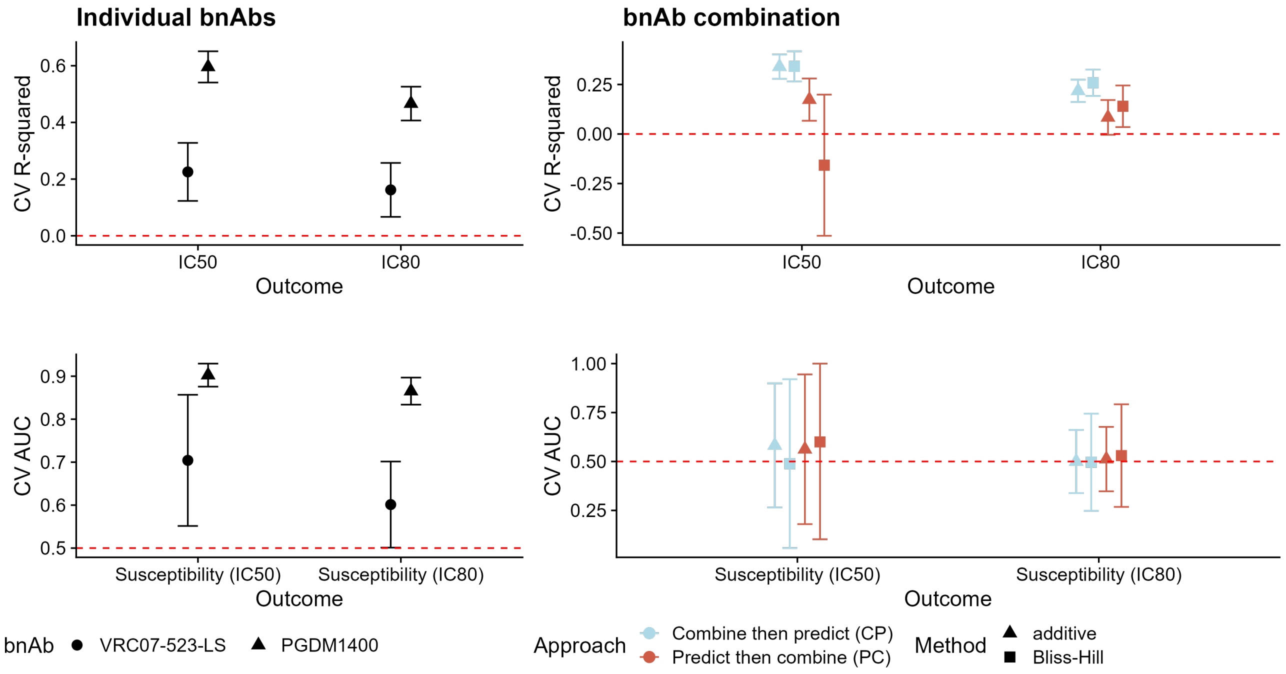

Figure 6: Prediction performance for continuous (top row, CV R-squared) and binary (bottom row, CV AUC) neutralization outcomes for individual bnAbs (left-hand column) and the combination (right-hand column) VRC07-523-LS + PGDM1400. For individual bnAbs, prediction performance is evaluated against the observed IC_{50} or IC_{80} values for the given bnAb; shapes denote the bnAb. For combination bnAbs, prediction performance is evaluated against the calculated combination IC_{50} or IC_{80} values based on the observed bnAb-specific values using the additive or Bliss-Hill method; shapes denote the combination method (additive or Bliss-Hill) and color denotes the approach (CP or PC). Error bars reflect 95% confidence intervals.

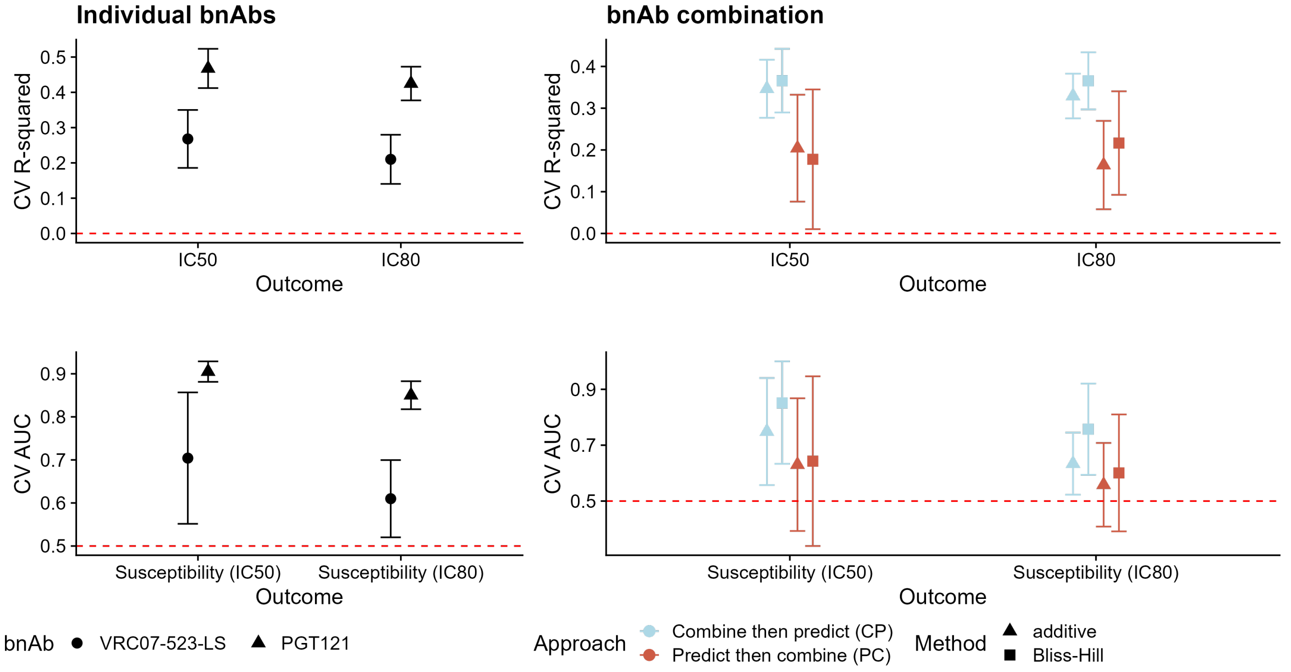

Figure 7: Prediction performance for continuous (top row, CV R-squared) and binary (bottom row, CV AUC) neutralization outcomes for individual bnAbs (left-hand column) and the combination (right-hand column) VRC07-523-LS + PGT121. For individual bnAbs, prediction performance is evaluated against the observed IC_{50} or IC_{80} values for the given bnAb; shapes denote the bnAb. For combination bnAbs, prediction performance is evaluated against the calculated combination IC_{50} or IC_{80} values based on the observed bnAb-specific values using the additive or Bliss-Hill method; shapes denote the combination method (additive or Bliss-Hill) and color denotes the approach (CP or PC). Error bars reflect 95% confidence intervals.

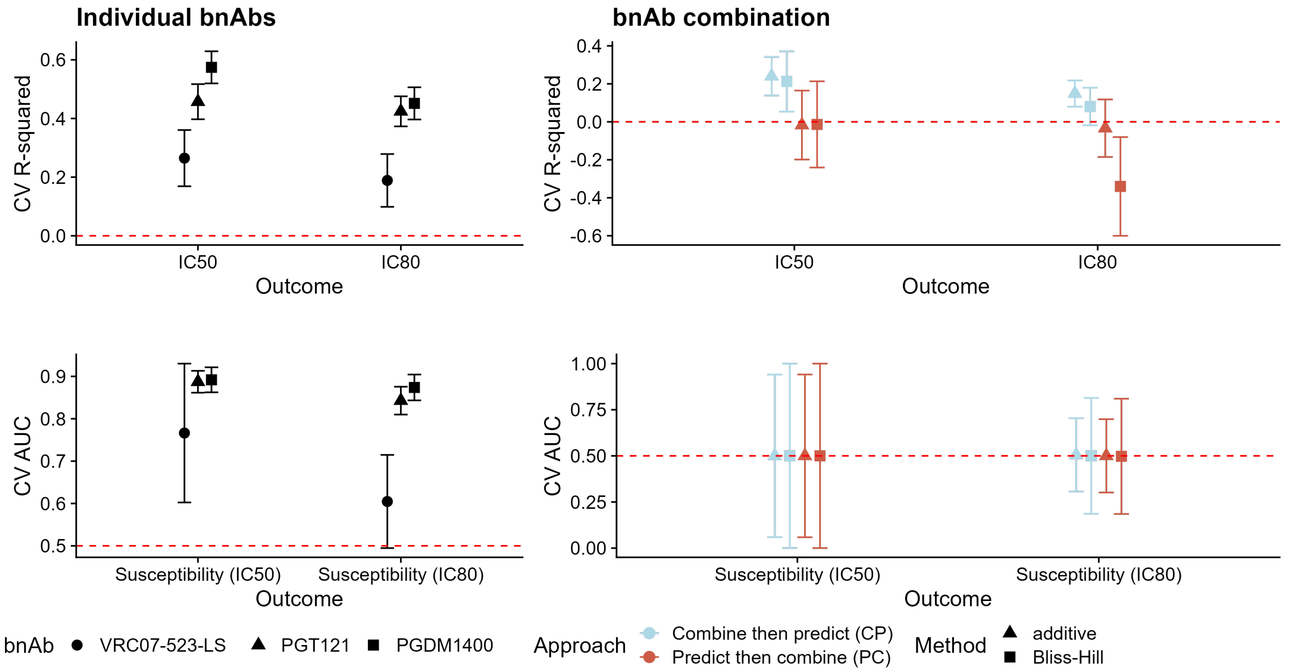

Figure 8: Prediction performance for continuous (top row, CV R-squared) and binary (bottom row, CV AUC) neutralization outcomes for individual bnAbs (left-hand column) and the combination (right-hand column) VRC07-523-LS + PGT121 + PGDM1400. For individual bnAbs, prediction performance is evaluated against the observed IC_{50} or IC_{80} values for the given bnAb; shapes denote the bnAb. For combination bnAbs, prediction performance is evaluated against the calculated combination IC_{50} or IC_{80} values based on the observed bnAb-specific values using the additive or Bliss-Hill method; shapes denote the combination method (additive or Bliss-Hill) and color denotes the approach (CP or PC). Error bars reflect 95% confidence intervals.

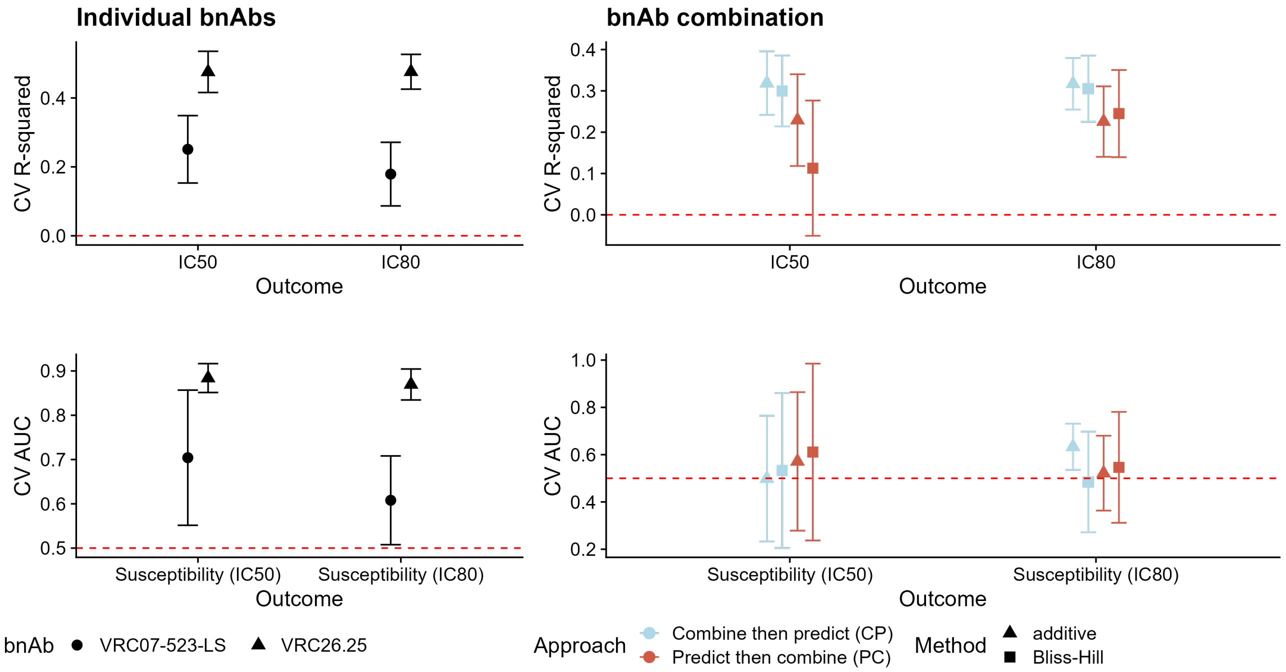

Figure 9: Prediction performance for continuous (top row, CV R-squared) and binary (bottom row, CV AUC) neutralization outcomes for individual bnAbs (left-hand column) and the combination (right-hand column) VRC07-523-LS + VRC26.25. For individual bnAbs, prediction performance is evaluated against the observed IC_{50} or IC_{80} values for the given bnAb; shapes denote the bnAb. For combination bnAbs, prediction performance is evaluated against the calculated combination IC_{50} or IC_{80} values based on the observed bnAb-specific values using the additive or Bliss-Hill method; shapes denote the combination method (additive or Bliss-Hill) and color denotes the approach (CP or PC). Error bars reflect 95% confidence intervals.

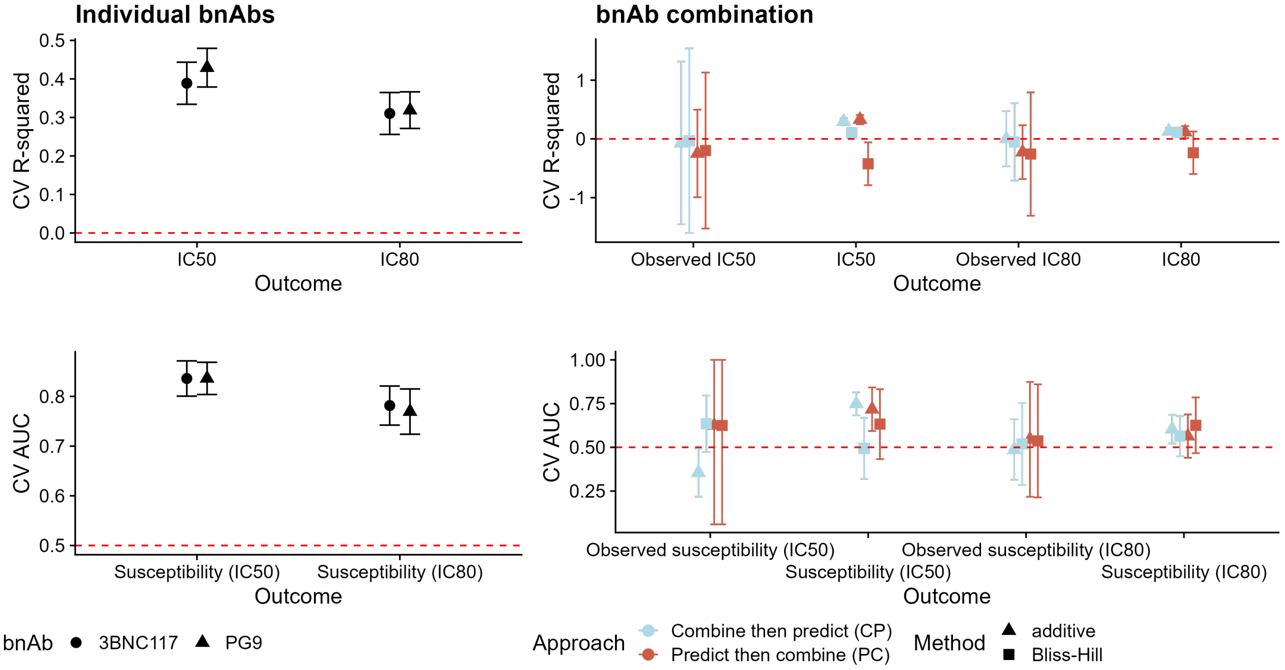

Figure 10: Prediction performance for continuous (top row, CV R-squared) and binary (bottom row, CV AUC) neutralization outcomes for individual bnAbs (left-hand column) and the combination (right-hand column) 3BNC117 + PG9. For individual bnAbs, prediction performance is evaluated against the observed IC_{50} or IC_{80} values for the given bnAb; shapes denote the bnAb. For combination bnAbs, prediction performance is evaluated against both the observed IC_{50} or IC_{80} values based on the bnAb regimen (denoted by the prefix "observed") and the calculated combination IC_{50} or IC_{80} values based on the observed bnAb-specific values using the additive or Bliss-Hill method; shapes denote the combination method (additive or Bliss-Hill) and color denotes the approach (CP or PC). Error bars reflect 95% confidence intervals.

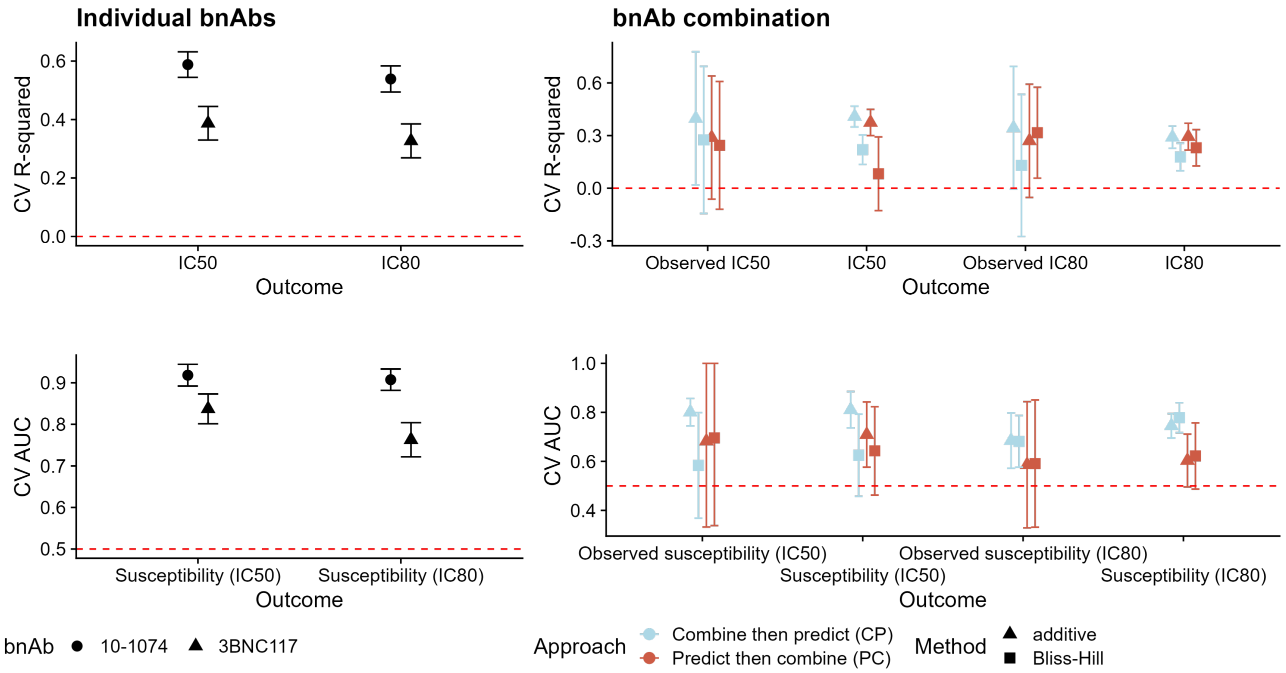

Figure 11: Prediction performance for continuous (top row, CV R-squared) and binary (bottom row, CV AUC) neutralization outcomes for individual bnAbs (left-hand column) and the combination (right-hand column) 10-1074 + 3BNC117. For individual bnAbs, prediction performance is evaluated against the observed IC_{50} or IC_{80} values for the given bnAb; shapes denote the bnAb. For combination bnAbs, prediction performance is evaluated against both the observed IC_{50} or IC_{80} values based on the bnAb regimen (denoted by the prefix "observed") and the calculated combination IC_{50} or IC_{80} values based on the observed bnAb-specific values using the additive or Bliss-Hill method; shapes denote the combination method (additive or Bliss-Hill) and color denotes the approach (CP or PC). Error bars reflect 95% confidence intervals.

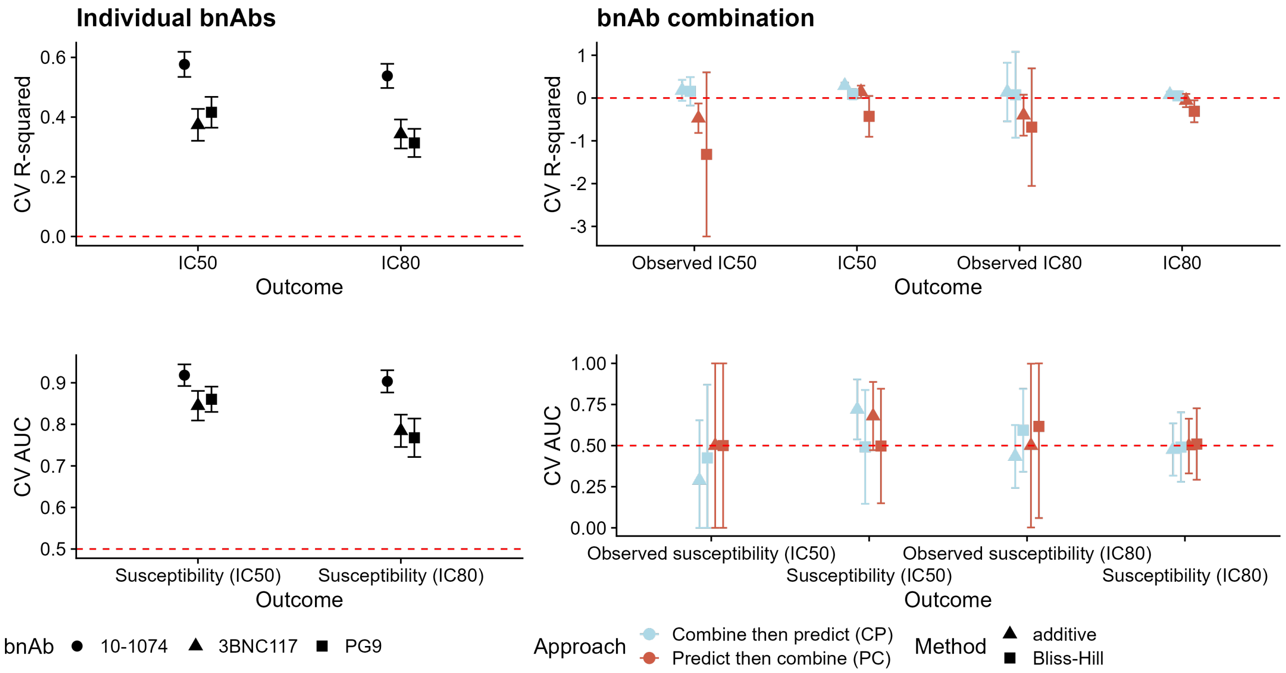

Figure 12: Prediction performance for continuous (top row, CV R-squared) and binary (bottom row, CV AUC) neutralization outcomes for individual bnAbs (left-hand column) and the combination (right-hand column) 10-1074 + 3BNC117 + PG9. For individual bnAbs, prediction performance is evaluated against the observed IC_{50} or IC_{80} values for the given bnAb; shapes denote the bnAb. For combination bnAbs, prediction performance is evaluated against both the observed IC_{50} or IC_{80} values based on the bnAb regimen (denoted by the prefix "observed") and the calculated combination IC_{50} or IC_{80} values based on the observed bnAb-specific values using the additive or Bliss-Hill method; shapes denote the combination method (additive or Bliss-Hill) and color denotes the approach (CP or PC). Error bars reflect 95% confidence intervals.

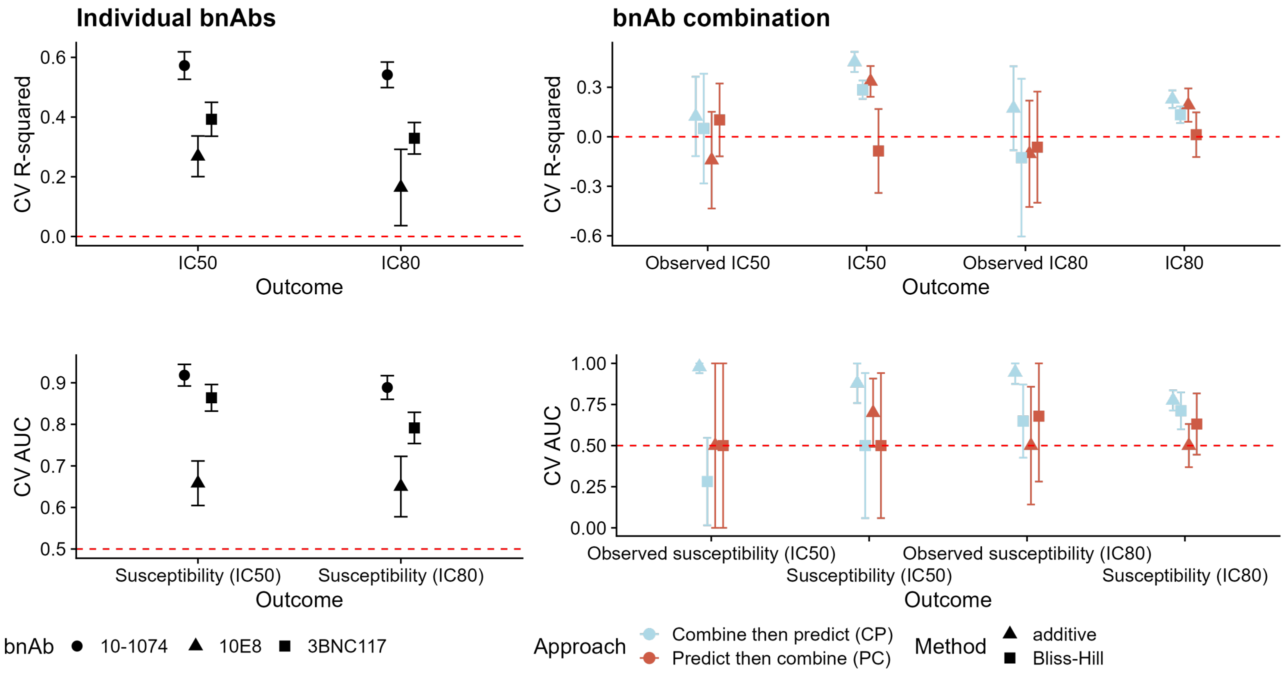

Figure 13: Prediction performance for continuous (top row, CV R-squared) and binary (bottom row, CV AUC) neutralization outcomes for individual bnAbs (left-hand column) and the combination (right-hand column) 10-1074 + 10E8 + 3BNC117. For individual bnAbs, prediction performance is evaluated against the observed IC_{50} or IC_{80} values for the given bnAb; shapes denote the bnAb. For combination bnAbs, prediction performance is evaluated against both the observed IC_{50} or IC_{80} values based on the bnAb regimen (denoted by the prefix "observed") and the calculated combination IC_{50} or IC_{80} values based on the observed bnAb-specific values using the additive or Bliss-Hill method; shapes denote the combination method (additive or Bliss-Hill) and color denotes the approach (CP or PC). Error bars reflect 95% confidence intervals.

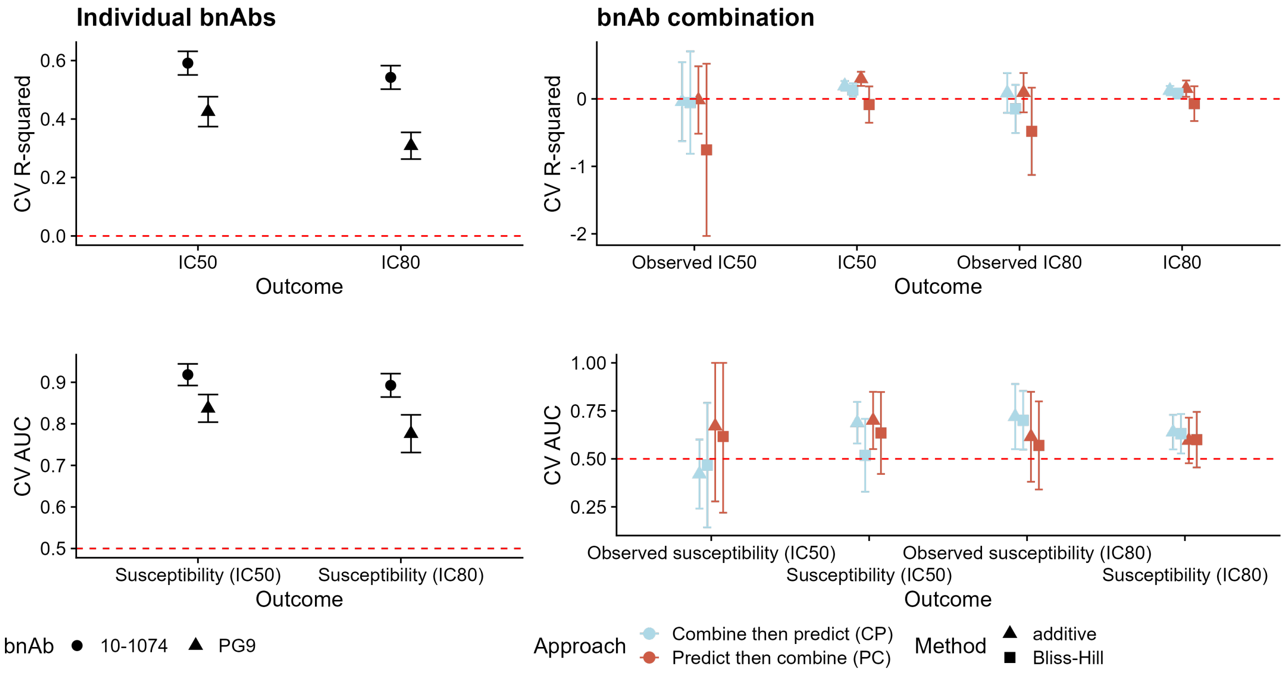

Figure 14: Prediction performance for continuous (top row, CV R-squared) and binary (bottom row, CV AUC) neutralization outcomes for individual bnAbs (left-hand column) and the combination (right-hand column) 10-1074 + PG9. For individual bnAbs, prediction performance is evaluated against the observed IC_{50} or IC_{80} values for the given bnAb; shapes denote the bnAb. For combination bnAbs, prediction performance is evaluated against both the observed IC_{50} or IC_{80} values based on the bnAb regimen (denoted by the prefix "observed") and the calculated combination IC_{50} or IC_{80} values based on the observed bnAb-specific values using the additive or Bliss-Hill method; shapes denote the combination method (additive or Bliss-Hill) and color denotes the approach (CP or PC). Error bars reflect 95% confidence intervals.

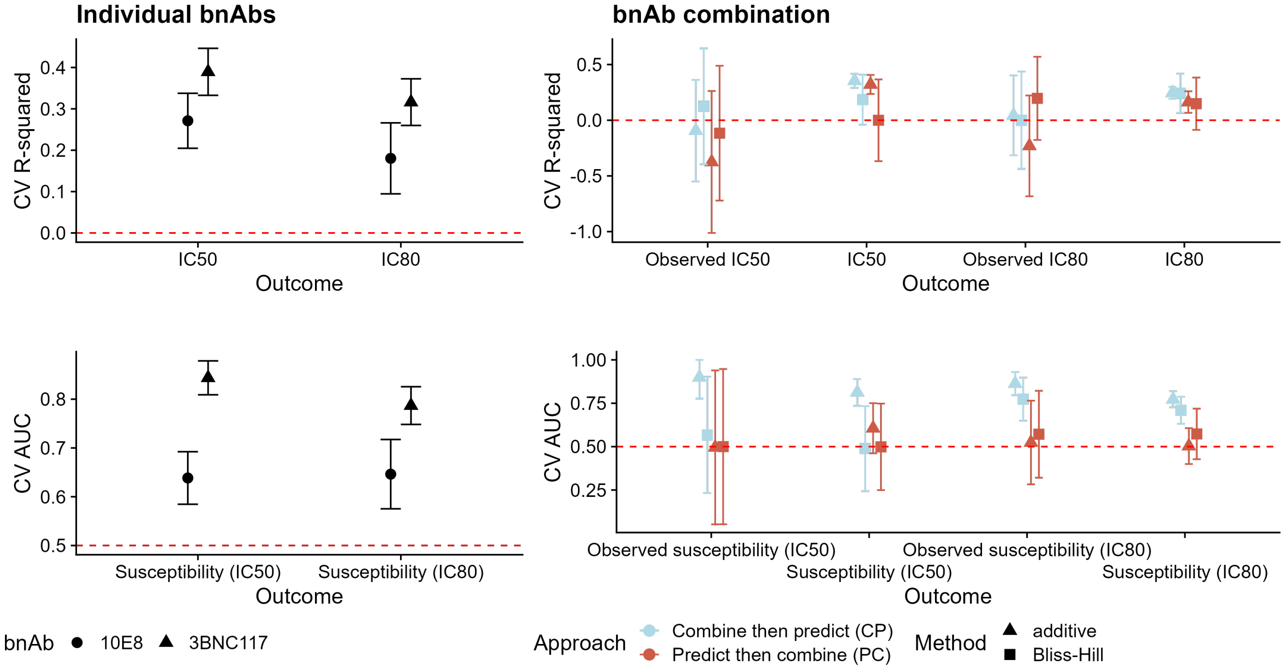

Figure 15: Prediction performance for continuous (top row, CV R-squared) and binary (bottom row, CV AUC) neutralization outcomes for individual bnAbs (left-hand column) and the combination (right-hand column) 10E8 + 3BNC117. For individual bnAbs, prediction performance is evaluated against the observed IC_{50} or IC_{80} values for the given bnAb; shapes denote the bnAb. For combination bnAbs, prediction performance is evaluated against both the observed IC_{50} or IC_{80} values based on the bnAb regimen (denoted by the prefix "observed") and the calculated combination IC_{50} or IC_{80} values based on the observed bnAb-specific values using the additive or Bliss-Hill method; shapes denote the combination method (additive or Bliss-Hill) and color denotes the approach (CP or PC). Error bars reflect 95% confidence intervals.

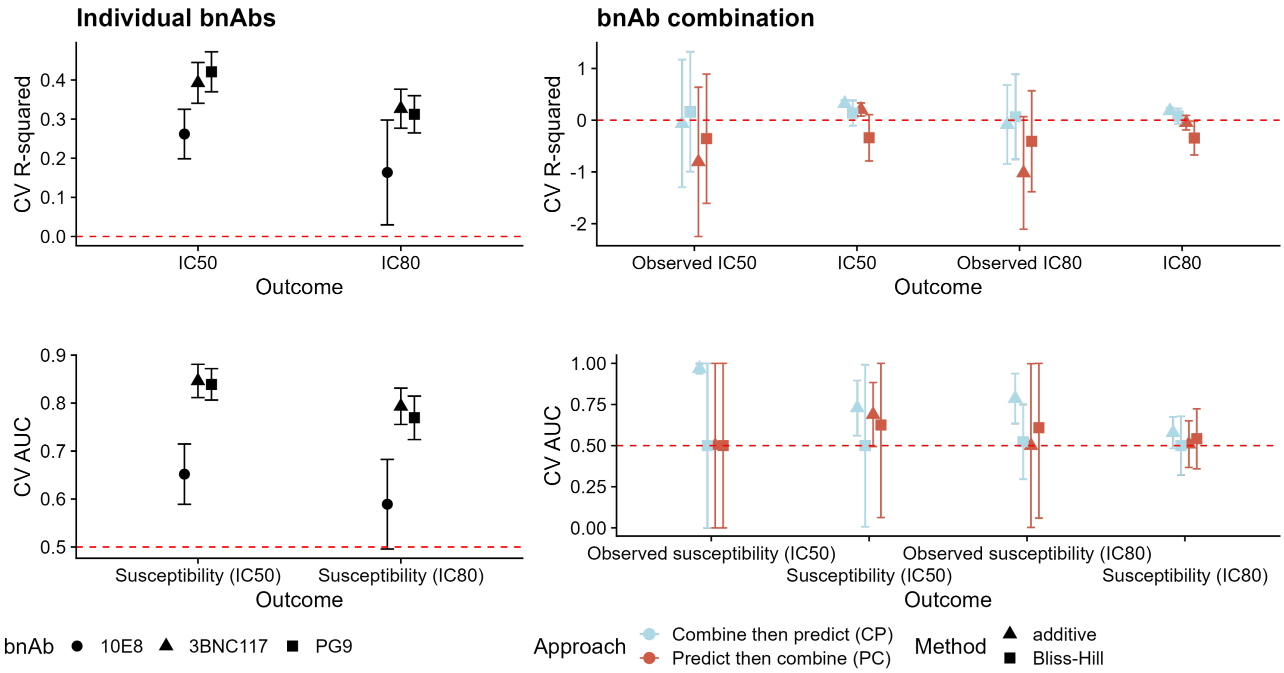

Figure 16: Prediction performance for continuous (top row, CV R-squared) and binary (bottom row, CV AUC) neutralization outcomes for individual bnAbs (left-hand column) and the combination (right-hand column) 10E8 + 3BNC117 + PG9. For individual bnAbs, prediction performance is evaluated against the observed IC_{50} or IC_{80} values for the given bnAb; shapes denote the bnAb. For combination bnAbs, prediction performance is evaluated against both the observed IC_{50} or IC_{80} values based on the bnAb regimen (denoted by the prefix "observed") and the calculated combination IC_{50} or IC_{80} values based on the observed bnAb-specific values using the additive or Bliss-Hill method; shapes denote the combination method (additive or Bliss-Hill) and color denotes the approach (CP or PC). Error bars reflect 95% confidence intervals.

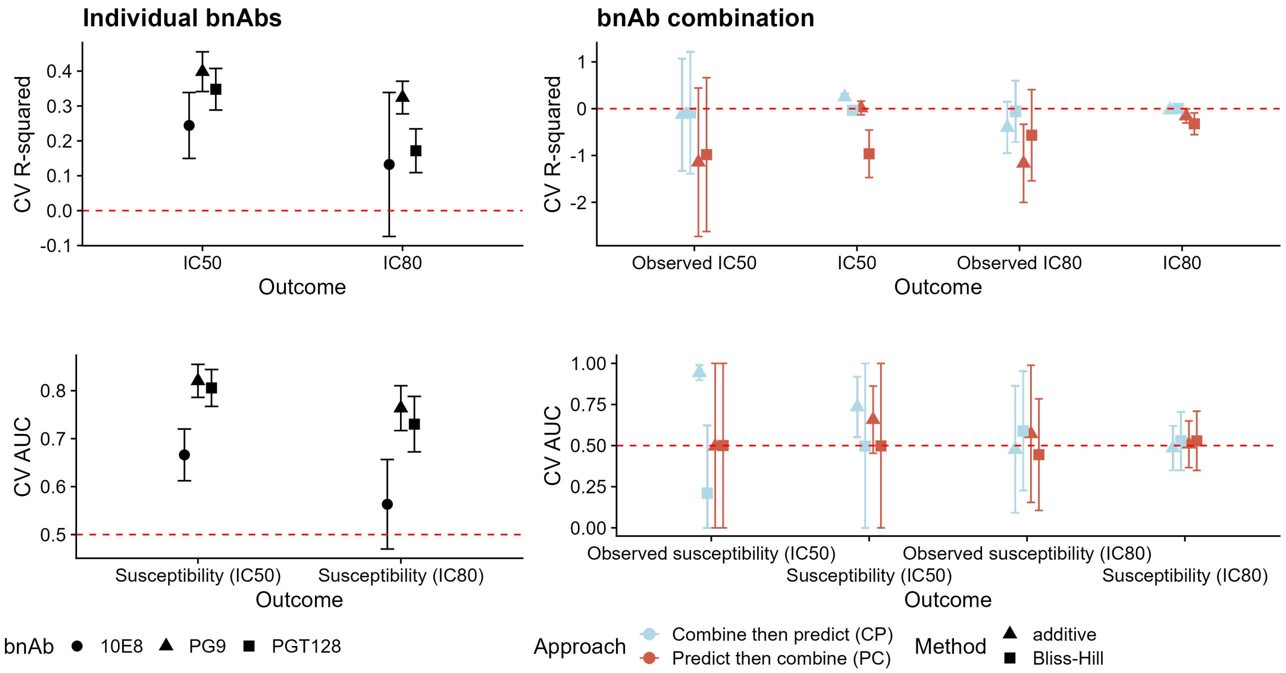

Figure 17: Prediction performance for continuous (top row, CV R-squared) and binary (bottom row, CV AUC) neutralization outcomes for individual bnAbs (left-hand column) and the combination (right-hand column) 10E8 + PG9 + PGT128. For individual bnAbs, prediction performance is evaluated against the observed IC_{50} or IC_{80} values for the given bnAb; shapes denote the bnAb. For combination bnAbs, prediction performance is evaluated against both the observed IC_{50} or IC_{80} values based on the bnAb regimen (denoted by the prefix "observed") and the calculated combination IC_{50} or IC_{80} values based on the observed bnAb-specific values using the additive or Bliss-Hill method; shapes denote the combination method (additive or Bliss-Hill) and color denotes the approach (CP or PC). Error bars reflect 95% confidence intervals.

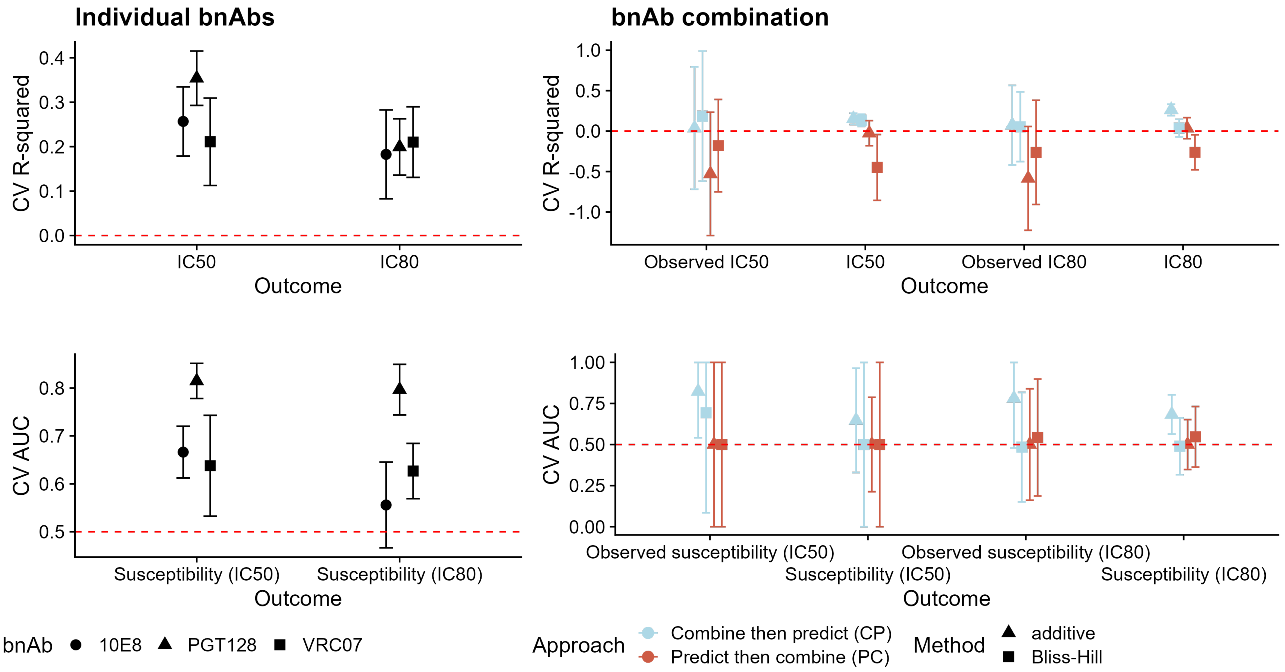

Figure 18: Prediction performance for continuous (top row, CV R-squared) and binary (bottom row, CV AUC) neutralization outcomes for individual bnAbs (left-hand column) and the combination (right-hand column) 10E8 + PGT128 + VRC07. For individual bnAbs, prediction performance is evaluated against the observed IC_{50} or IC_{80} values for the given bnAb; shapes denote the bnAb. For combination bnAbs, prediction performance is evaluated against the calculated combination IC_{50} or IC_{80} values based on the observed bnAb-specific values using the additive or Bliss-Hill method; shapes denote the combination method (additive or Bliss-Hill) and color denotes the approach (CP or PC). Error bars reflect 95% confidence intervals.

Table 1: Broadly neutralizing antibody combination regimens either with at least 125 sequences in CATNAP with direct measurement of combination neutralization or undergoing HVTN/HPTN clinical testing as of October 2022, along with the proportion susceptible to each bnAb in the combination (defined as $IC_{80} < 1$ μg/mL). Individual-bnAb susceptibility proportions are listed in order of the bnAbs within the combination. Combination susceptibility proportions are listed in order of additive, Bliss-Hill, and directly-measured combination susceptibility (if available).

Combination regimen	Proportion susceptible to each bnAb	Proportion susceptible to the combination
BG1 + BG18 + NC37	9.24%, 44.54%, 10.17%	50.85%, 58.47%, 47.9%
PG9 + PGT128	46.52%, 47%	73.33%, 82%, 81.6%
PG9 + PGT128 + VRC07	46.52%, 47%, 61.22%	86.62%, 90.97%, 94.4%
PG9 + VRC07	46.52%, 61.22%	77.55%, 88.27%, 88.8%
PGT128 + VRC07	47%, 61.22%	75.92%, 88.29%, 85.6%
VRC07-523-LS + 10-1074	78.75%, 48.89%	89.5%, 93.5%
VRC07-523-LS + PGDM1400	78.75%, 49.52%	89.75%, 96%
VRC07-523-LS + PGT121	78.75%, 46.68%	86.75%, 92.75%
VRC07-523-LS + PGDM1400 + PGT121	78.75%, 46.68%, 49.52%	93.5%, 97.5%
VRC07-523-LS + VRC26.25	78.75%, 48%	88.5%, 94.75%
3BNC117 + PG9	61.95%, 46.52%	79.25%, 87.25%, 91.2%
10-1074 + 3BNC117	48.89%, 61.95%	81.18%, 86.9%, 84.8%
10-1074 + 3BNC117 + PG9	48.89%, 61.95%, 46.52%	90.48%, 94.49%, 96.8%
10-1074 + 10E8	48.89%, 21.34%	59.65%, 79.7%, 64.8%
10-1074 + 10E8 + 3BNC117	48.89%, 21.34%, 61.95%	83.21%, 91.23%, 93.6%
10-1074 + PG9	48.89%, 46.52%	77.19%, 86.72%, 80%
10E8 + 3BNC117	21.34%, 61.95%	66.25%, 86.5%, 83.2%
10E8 + 3BNC117 + PG9	21.34%, 61.95%, 46.52%	85.75%, 91.75%, 96.8%
10E8 + PG9 + PGT128	21.34%, 46.52%, 47%	79.67%, 89%, 94.4%
10E8 + PG9 + VRC07	21.34%, 46.52%, 61.22%	86.22%, 90.31%, 96.8%
10E8 + PGT128 + VRC07	21.34%, 47%, 61.22%	83.28%, 88.63%, 92.8%
